# Fairness-aware artificial intelligence tutoring for multilingual learners: evaluating adaptive feedback across L1 and proficiency groups

**DOI:** 10.3389/frai.2026.1934329

**Published:** 2026-09-22

**Authors:** Patrick O. Akinwumi, Meihua Qian

**Affiliations:** College of Education, Clemson University, Clemson, SC, United States

**Keywords:** adaptive learning systems, artificial intelligence in language learning, automated writing evaluation (AWE), multilingual learners, second language acquisition (SLA)

## Abstract

**Introduction:**

Recent advances in large language models (LLMs), such as GPT-4, have created new opportunities for intelligent computer-assisted language learning, particularly through personalized feedback for multilingual English learners. Building on research in automated writing evaluation (AWE), this study developed an equity-aware AI tutoring framework that integrates GPT-generated feedback with rule-based simulated feedback for learner spelling errors. The framework draws on annotated TOEFL-Spell data enriched with learner first-language (L1) and English proficiency information to examine feedback quality, accessibility, personalization, and potential subgroup disparities.

**Methods:**

A verified matched analysis of 32 complete GPT-simulated feedback pairs was conducted using feedback length, Flesch Reading Ease, BERTScore, non-parametric subgroup tests, paired comparisons, effect-size estimates, and exploratory regression models controlling for selected input characteristics.

**Results:**

GPT-generated feedback demonstrated substantial semantic correspondence with simulated feedback, achieving an overall BERTScore precision of 0.8370, recall of 0.8742, and F1 score of 0.8552. GPT feedback was significantly longer than simulated feedback (*M* = 608.12 vs. 218.69 characters, *p* < 0.001) and, contrary to the preliminary analysis, was also significantly more readable on average (*M* = 75.62 vs. 64.64, *p* < 0.001). GPT feedback length did not differ significantly across L1 (*p* = 0.143) or proficiency groups (*p* = 0.422), while GPT readability showed a significant omnibus difference across proficiency levels (*p* = 0.047); however, no pairwise comparison remained significant after Holm correction. BERTScore F1 was stable across both L1 (*p* = 0.775) and proficiency groups (*p* = 0.500). Exploratory controlled analyses likewise provided no consistent evidence that L1 independently predicted feedback readability or semantic alignment after accounting for prompt and error characteristics.

**Discussion:**

Accordingly, observed subgroup variation is interpreted as descriptive heterogeneity rather than definitive evidence of algorithmic or sociolinguistic bias. Because L1 and proficiency were not fully crossed in the analytical sample, their independent effects could not be completely disentangled. A Gradio-based interface (https://huggingface.co/spaces/Oluyori/equity-ai-tutor) was additionally developed to demonstrate the practical deployment of the tutoring framework. The findings demonstrate LLM-based feedback potential for semantically aligned and accessible language support while emphasizing the need for larger, balanced samples, learner-centered evaluation, and multidimensional fairness assessment before claims regarding equitable educational performance can be made.

## 1 Introduction

Education is inherently a multilingual and multicultural endeavor, so is human language is complex and often ambiguous, with AI technologies showing promising potential in improving learner's experiences, and educational systems globally (Gligorea et al., 2023; Gupta et al., 2025). Recent research in artificial intelligence (AI) and education underscores the transformative potential of intelligent tutoring systems (ITS) and adaptive learning platforms to personalize instruction and feedback according to diverse learners' needs (Chinta et al., 2024; Lin et al., 2023). AI-driven learning analytics, for instance, can customize educational pathways for individual students, thereby enhancing both learning efficiency and academic outcomes (Lin et al., 2023; Singh et al., 2022). In parallel, automated grading systems offer consistent evaluation through standardized rubrics, reducing human error and alleviating teacher workload (Chinta et al., 2024). Natural language processing (NLP) applications, though, even the most advanced NLP algorithms make errors, further support multilingual learners by mitigating language barriers.

Despite the growing capabilities of AI-powered educational technologies, ensuring equitable outcomes for multilingual learners (MLs) remains a concern. Although intelligent tutoring systems (ITS) such as AutoTutor and Carnegie Learning have demonstrated efficacy in adapting to individual learning paces and cognitive styles (Graesser et al., 2005; Nye et al., 2014), they frequently underperform for students from linguistically diverse backgrounds due to limited cultural and linguistic adaptability. These limitations are rooted in a reliance on monolingual training corpora and standardized linguistic assumptions, which can hinder comprehension, engagement, and learning outcomes for MLs (Heffernan and Heffernan, 2014; Warschauer and Matuchniak, 2010).

Recent efforts to incorporate natural language processing (NLP) tools for non-native speakers represent an encouraging shift, yet they often neglect broader systemic issues such as digital equity, language ideology, and cultural responsiveness. Moreover, many equity-focused learning systems emphasize access over interactional granularity, lacking differentiated strategies that align with learners' linguistic repertoires (Paris and Alim, 2017). These shortcomings reflect a broader trend in AI in education (AIEd), where equity considerations remain peripheral. For instance, most ITS and AIEd evaluations have not explicitly addressed fairness or algorithmic bias (Létourneau et al., 2025). A recent systematic review found no studies in K-12 settings that examined the ethical or fairness implications of AI tutors (Létourneau et al., 2025).

As AI-powered translation and language assistance tools make educational content accessible to non-native speakers, empowering learners globally to engage with materials in their own languages (Babaci-Wilhite et al., 2025). These multilingual capabilities are increasingly recognized as essential to fostering equity in education. The literature on equitable AI emphasizes the importance of cultural and linguistic inclusivity in educational technologies.

Davoodi (2024) proposes the EQUAL AI framework, which integrates linguistic support, cultural representation, and inclusivity into AI-enhanced liberal arts instruction for multilingual students (Davoodi, 2024). Similarly, Babaci-Wilhite et al. (2025) argue that AI and NLP tools can amplify multilingual and multicultural assets in the classroom, promoting both equity and pedagogical innovation by aligning technology with students' cultural identities (Babaci-Wilhite et al., 2025). Systematic reviews of ITS literature reinforce these findings, highlighting core instructional strategies such as adaptive scaffolding, immediate feedback, and differentiated learning tasks as effective supports for diverse student populations (Atapattu and Falkner, 2016; Ogunkunle and Qu, 2020). Collectively, this body of research illustrates how AI in education, through personalized instruction and multilingual support, can enhance learning outcomes, particularly when ITS are designed with an awareness of students' linguistic and cultural backgrounds (Davoodi, 2024).

In NLP and large language model (LLM) research, fairness assessments have overwhelmingly concentrated on English, while other languages and cultural contexts receive significantly less attention (Ramesh et al., 2023). This imbalance has tangible consequences for education: English-centric AI models risk privileging speakers of dominant languages while marginalizing others, thereby perpetuating or even exacerbating existing inequities (Ahuja K. et al., 2023; Ahuja S. et al., 2023; Lai et al., 2023). These findings underscore the need for equity-aware design approaches that centre linguistic and cultural inclusivity from the outset.

To address these challenges, this study proposes the development of an *Equity-Aware AI Tutoring Engine* that integrates fairness-oriented algorithms with culturally responsive pedagogy. Drawing on socio-technical frameworks and fairness-aware machine learning paradigms (Binns, 2018; Holstein et al., 2019), the proposed system personalizes content delivery based not only on learners' proficiency but also on their linguistic backgrounds. Specifically, NLP-based feedback generation incorporates learners' L1 backgrounds and proficiency levels, while fairness-oriented evaluation examines potential disparities in feedback characteristics across learner groups (Davoodi, 2024; Ramesh et al., 2023).

This research aligns with emerging calls to address algorithmic bias and promote inclusive practices in educational AI (Davoodi, 2024; Ramesh et al., 2023). Frameworks like EQUAL AI offer a foundation for integrating linguistic support, cultural representation, and fairness metrics into system design, supporting the development of tutoring technologies that are both equitable and educationally effective (Davoodi, 2024; Ramesh et al., 2023). By embedding learner-sensitive feedback and equity-oriented evaluation into the core framework, this project aims to move beyond access-focused solutions, advancing an approach to AI-driven education that places language equity and social justice at its centre (Yilmaz, 2021).

## 2 Literature review

### 2.1 AI-enhanced personalization in education

The field of Artificial Intelligence in Education (AIED) has grown rapidly in response to calls for scalable, high-quality, and inclusive education. Adaptive learning platforms, intelligent tutoring systems (ITS), and AI-based analytics tools are increasingly used to personalize instruction for individual learners (Godwin-Jones, 2012; Lin et al., 2023). These systems dynamically adjust content difficulty, sequence tasks based on learner performance and forecast outcomes to inform instruction. For instance, adaptive learning platforms curate learning paths matched to students' proficiency, while NLP-powered chatbots offer on-demand language practice and academic support (Létourneau et al., 2025).

Central to this progress are ITSs, defined as computer-based environments that model students' knowledge and deliver adaptive feedback in real time. Pioneering work e.g., (Carbonell, 1970) introduced err or diagnosis and sequencing algorithms, which modern ITSs have since expanded into sophisticated learner models encompassing cognition, motivation, and metacognition. According to Lin et al. (2023), these systems “use artificial intelligence … to provide personalized and adaptive instruction,” leveraging real-time data to select appropriately challenging tasks (Lin et al., 2023; VanLehn, 2011). A popular example is Duolingo, which personalizes language learning through ITS methods. As noted by UNESCO, ITSs represent some of the most extensively studied AI tools in education (Miao and Holmes, 2021).

### 2.2 NLP for learner error correction

Natural Language Processing (NLP), a subfield of computer science and artificial intelligence, enables computers to understand and interpret human language, both written and spoken, much like humans do. It ha**s** become a foundational tool in language education, particularly for automated feedback and assessment. Recent surveys categorize educational NLP tasks into areas such as question answering, automated scoring, and error correction (Lan et al., 2024). Among these, error correction systems, often embedded in Automated Writing Evaluation (AWE) tools, offer immediate feedback on grammar, spelling, and textual coherence. Widely used applications like Grammarly exemplify the real-world adoption of such tools, with Lan et al. (2024) explicitly identifying Grammarly as a mainstream educational NLP system.

Modern NLP approaches have shifted from rule-based and statistical models to deep learning architectures, particularly Transformer-based systems. These models significantly outperform earlier techniques in handling complex linguistic inputs and generating context-aware feedback (Zheng and Zhang, 2025). Study by Zheng and Zhang (2025) describe a Transformer-powered writing assistant that analyzes grammar, vocabulary, syntax, and discourse coherence to provide multidimensional feedback (Asad et al., 2024; Dutta et al., 2024). Such systems adapt to diverse writing genres and offer more nuanced guidance than traditional grammar checkers.

The pedagogical benefits of NLP-driven feedback are substantial. Automated systems reduce teacher workload, enable unlimited student practice, and facilitate scalable assessment, particularly in large-scale settings like MOOCs (Zheng and Zhang, 2025; Zomer and Frankenberg-Garcia, 2021). Importantly, sequence-to-sequence models trained on learner corpora can be adapted to specific L1 backgrounds, enabling corrections that account for interlanguage features and recurrent learner-specific errors (Zomer and Frankenberg-Garcia, 2021). These L1-sensitive models help capture linguistic patterns that generic GEC (Grammatical Error Correction) tools often miss.

Nonetheless, current systems have limitations. Many focus narrowly on surface-level errors, neglecting higher-order dimensions like organization, argumentation, or style (Zheng and Zhang, 2025; Zomer and Frankenberg-Garcia, 2021). Even state-of-the-art neural models may struggle with non-standard syntax or long-range dependencies, a challenge noted in earlier RNN-based systems and still relevant for some Transformer implementations (Zheng and Zhang, 2025). Moreover, large-scale models such as GPT-3.5, while powerful, pose concerns around opacity, data privacy, computational costs, and lack of educational tuning (Zheng and Zhang, 2025). A further gap lies in interpretability: most systems provide corrections without pedagogically meaningful explanations, limiting their instructional value (Lan et al., 2024).

Commercial tools like Grammarly, ProWritingAid, and automated essay scoring platforms offer accessible error correction but prioritize correctness over learning. As Zheng and Zhang argue, these tools “offer reliable basic grammar correction but lack adaptability to educational contexts,” often operating as closed systems with fixed rule sets and limited transparency (Zheng and Zhang, 2025). They typically do not adjust to learners' proficiency levels or L1 backgrounds, nor do they provide metacognitive scaffolding. While widely adopted, commercial tools are rarely the focus of academic evaluation, only about 10% of studies directly assess them, leaving a substantial research emphasis on developing open-source, pedagogically aligned NLP models (Lan et al., 2024; Zheng and Zhang, 2025).

### 2.3 Fairness and bias in educational AI

The integration of AI into educational settings introduces urgent concerns about fairness and bias. While AI-driven systems can personalize instruction and automate assessment, they also risk reinforcing existing inequities. As noted in recent reviews,

“AI systems can inadvertently perpetuate and amplify existing biases present in the data they are trained on, leading to unfair outcomes, particularly for historically marginalized groups.” (Chinta et al., 2024)

For instance, automated essay scoring models trained predominantly on native English speakers may favor dominant linguistic styles, systematically disadvantaging English Language Learners (ELLs) and students from underrepresented backgrounds (Jourdain and Sharma, 2016; Weigle, 2013).

Adaptive learning technologies may also reflect bias if past performance data, used to calibrate difficulty, are skewed. Chinta et al. (2024) categorize the primary sources of bias in educational AI as *data bias, algorithmic bias, and interaction bias*, and emphasize that intelligent tutoring systems must be designed to be

“fair, equitable, transparent, and beneficial to learners.” (Ali et al., 2023; Holmes et al., 2022)

Empirical evidence supports these concerns. Guo et al. (2025) found that scoring models trained primarily on non-ELL data exhibited significant disparities when evaluating ELL student responses, due to the unique linguistic features and rhetorical structures present in their writing. In science assessments, Guo et al. also demonstrated that such disparities diminished when ELLs were adequately represented in the training set, reinforcing the importance of balanced datasets (LeClair et al., 2009; Wilson and Huang, 2024). Similar patterns of bias have been observed across other demographics, including race and gender (Latif et al., 2023; Li et al., 2024).

Efforts to mitigate these biases are emerging. Strategies include *data augmentation, fairness-aware learning algorithms, post-processing adjustments*, and *evaluation frameworks* such as counterfactual fairness and equalized odds (Chinta et al., 2024; Wang et al., 2023). Tools like *Fairlearn* and *FairAWARE* have been developed to measure and reduce algorithmic unfairness. However, fairness interventions often involve trade-offs: improving group fairness can sometimes reduce overall model accuracy (Wang et al., 2023).

Ethical AI frameworks increasingly call *for transparency, explainability*, and *accountability*, particularly in high-stakes educational contexts (Adams et al., 2023; Ali et al., 2023; Holmes et al., 2022). Yet, most educational AI systems, especially commercial ones, lack transparency and do not account for learner demographics. Even in academic research, fairness is often under-addressed. Chinta et al. (2024) argue that truly equitable AI in education requires interdisciplinary strategies, which remain largely absent from current systems.

The literature identifies clear risks of bias in AI-powered educational tools, especially in assessment. While fairness-aware methodologies are gaining traction, few systems fully implement them. Addressing this gap is essential: biased AI undermines learner trust and can exacerbate achievement disparities. Recent scholarship highlights the need to treat fairness not as an afterthought but as a foundational design principle in educational AI (Chinta et al., 2024).

### 2.4 Multilingual learner assessment and L1-specific challenges

As classrooms become increasingly multilingual, a critical gap emerges in AI-driven tutoring and assessment systems: most are developed using English-dominant datasets and fail to accommodate first-language (L1) influences. Research has shown that learners' native languages significantly shape their writing patterns, especially through transfer effects, which standard error-correction models may overlook. Zomer and Frankenberg-Garcia (2021) note that few studies explicitly address L1 transfer in writing, despite its importance in understanding learner error patterns. Their work demonstrates that writing-correction models fine-tuned on L1-augmented data, such as simulating English writing by L1-Portuguese speakers, perform better at identifying L1-specific fluency issues than generic grammatical error correction (GEC) tools (Zomer and Frankenberg-Garcia, 2021).

The challenge extends beyond writing. Large language models (LLMs) trained primarily on English exhibit significant performance disparities across under-resourced languages. Gupta et al. (2025), for instance, evaluated popular LLMs across six non-English languages (Hindi, Arabic, Farsi, Telugu, Ukrainian, Czech) on tasks like feedback generation and grading. Their findings revealed consistent performance drops for low-resource languages, with outcomes correlating closely to the representation of each language in the training data (Gupta et al., 2025). This raises equity concerns, as AI tutors built on such models may systematically advantage English-speaking learners while underserving others.

Beyond model performance, many AI systems do not support multilingual input practices such as code-switching or L1 glosses, which are common among language learners. Most automated essay scoring tools, for example, expect monolingual responses in the target language, ignoring culturally or linguistically relevant annotations. As Chinta et al. (2024) argue, equitable education requires robust translation tools and L1-aware supports, yet “AI-driven language translation services” remain underutilized in educational contexts. Bias analyses by Guo et al. (2025) further show that AI scoring systems penalize ELLs when strong L1 influences are present, especially in complex tasks like science explanation, suggesting that existing models may misinterpret L1-based discourse structures as errors (Guo et al., 2025).

While the literature acknowledges the unique challenges faced by multilingual learners, most existing ITS and NLP systems remain ill-equipped to address them. Few systems are designed to adapt to learners' linguistic backgrounds, and NLP models overwhelmingly focus on English or a few major languages. This lack of linguistic inclusivity not only limits feedback accuracy but also risks mislabeling valid L1-based expressions as incorrect. To move toward educational equity, the field must invest in diverse learner corpora, develop L1-sensitive correction models, and adopt pedagogically grounded techniques that honor learners' full linguistic repertoires (Guo et al., 2025).

#### 2.4.1 Challenges facing multilingual learners

The definition of bilingualism depends on the underlying language ideology (Yilmaz, 2021). Bilingualism is conceptualized differently depending on the underlying language ideology, with two dominant perspectives: monoglossic and heteroglossic. The monoglossic view [also called the fractional or monolingual ideology; Grosjean (1989) defines bilingualism as the mastery of two separate languages, often evaluated through monolingual norms that value language separation and discourage code-switching or code-mixing (Blommaert, 2005, 2010; Lin, 2013)]. This ideology supports diglossic arrangements that privilege one “standard” language in educational settings, often leading to subtractive bilingualism where minoritized languages and identities are devalued (Abello-Contesse et al., 2013; Aboud and Meade, 1974; Cummins, 2005; De Jong, 2011; Ferguson, 1959; Fishman, 1967). In contrast, the heteroglossic perspective (also called the holistic or unitary view; Grosjean, 1989; Otheguy et al., 2015) challenges monolingual norms by viewing bilingualism as a fluid, dynamic integration of linguistic resources used contextually and strategically (Bakhtin, 2010; Grosjean, 1989; Pennycook, 2010). This ideology underpins models such as dynamic bilingualism, which recognize bilinguals' flexible and strategic use of language as normative and valuable in multilingual societies (Cenoz and Gorter, 2011; García, 2011). Rather than adhering to rigid linguistic boundaries, this approach positions translanguaging and the blending of linguistic repertoires as essential to meaning-making in diverse educational contexts (Garcìa et al., 2014; Lewis et al., 2012).

However, bias is especially problematic for multilingual learners (MLs), who often study in a language different from their home language. AIED systems typically lack linguistic sensitivity, treating all users as monolingual or native speakers. Research in educational measurement has long emphasized that assessments must measure constructs equivalently across languages and cultures to ensure validity and fairness (Ercikan and Por, 2020).

Studies confirm that AI systems trained predominantly on native English speakers can underperform on inputs from English Language Learners (ELLs). For example, Guo et al. (2025) found systematic scoring gaps in automated assessments, where models trained on imbalanced corpora performed worse on responses from ELLs (Guo et al., 2025). Similarly, Lewis (2025) identified cultural bias in language-learning apps, leading to significantly lower participation by minority students (Lewis, 2025).

#### 2.4.2 Teacher roles and pedagogical integration

Rather than replacing educators, ITSs can complement human instruction. Research indicates that AI tutors provide differentiated instruction and automate routine tasks such as grading and remediation, thereby freeing teachers to focus on higher-order pedagogy (Lin et al., 2023; Ogunkunle and Qu, 2020). Multiple studies document how automated feedback, scaffolding, and personalization through AI systems increase student engagement and instructional responsiveness beyond what traditional methods offer (Lin et al., 2023; Létourneau et al., 2025).

#### 2.4.3 Evidence of effectiveness and design conditions

Despite widespread enthusiasm, the empirical evidence on ITS effectiveness remains mixed. Systematic reviews reveal inconsistent outcomes depending on implementation fidelity, pedagogical design, and learner context. Létourneau et al. (2025) report that stakeholders struggle to navigate the gap between AI's potential to reduce inequities and its actual impact in classrooms (Létourneau et al., 2025). Earlier work by Kulik and Fletcher (2016) found that while some ITSs produced substantial gains (equivalent to an additional year of schooling), others showed negligible effects unless properly embedded within well-designed learning environments.

#### 2.4.4 Bias and fairness in AI systems

One of the most pressing concerns in educational AI is fairness. Research in computational linguistics and NLP highlights that large language models (LLMs) can absorb and reproduce biases present in their training data, including stereotypes and exclusionary language (Chinta et al., 2024; Gallegos et al., 2024). In education, such biases can be particularly harmful. For instance, automated essay scoring tools have been shown to rate essays by Black students lower than those by White students, even when the content quality is comparable, a disparity attributed to biased training data (Alvero et al., 2020; Chinta et al., 2024; Van Busum and Fang, 2023). Chinta et al. (2024) warn that AIED systems, without bias mitigation strategies, may unintentionally disadvantage certain student demographics (Chinta et al., 2024).

### 2.5 Emerging opportunities with generative AI

Despite these challenges, generative AI offers promising opportunities for greater inclusivity. Arslan (2024) suggest that LLMs can adapt learning content and assessment items to align with students' linguistic and cultural contexts, potentially improving accessibility and engagement for diverse learners (Arslan, 2024; Kasneci et al., 2023). Yet, these technologies also introduce new fairness risks related to content validity and representativeness (Kasneci et al., 2023).

### 2.6 Related work

Recent advances in educational AI, particularly large language models (LLMs) like GPT, Gemini, and LLaMA, have significantly expanded the scope of personalized instruction, real-time feedback, and interactive tutoring (Achiam et al., 2023; Leon, 2024; Mosher et al., 2024; Rooein et al., 2024; Roumeliotis et al., 2023). Early studies report that these models can scaffold learning and assist teachers (Alqahtani et al., 2023; Kasneci et al., 2023). However, the foundational training data for most LLMs remain predominantly English-centric despite claims of multilingualism (Radford et al., 2019; Ruder et al., 2022). As a result, these tools risk privileging dominant language learners and marginalizing those from underrepresented linguistic backgrounds, thereby exacerbating educational inequities (Ahuja K. et al., 2023; Ahuja S. et al., 2023; Lai et al., 2023).

The multilingual educational LLM benchmark released by ETH Zurich (Gupta et al., 2025; Roumeliotis et al., 2023) underscores this issue: across 91,000 LLM-generated outputs in six languages, significant performance disparities were observed, particularly for low-resource languages. This gap is highly consequential in education, where LLMs are increasingly integrated into high-stakes learning contexts without robust cross-linguistic evaluation (Alhafni et al., 2024; Raheja et al., 2023). These findings align with Mushtaq et al. (2025), who warn that culturally or linguistically misaligned AI-generated content may cause harm or reinforce exclusion in classroom environments.

In the broader landscape of *NLP in education*, Lan et al. (2024) categorize core applications such as question answering, automated assessment, and error correction. While these tools offer scalability and efficiency, many current systems lack interpretability, adaptability, and pedagogical alignment. Particularly, commercial systems like Grammarly prioritize surface-level accuracy over metacognitive feedback and are not tailored to L1-specific learner needs (Zheng and Zhang, 2025). Moreover, existing ITS models seldom account for sociolinguistic diversity or equity-based evaluation, as noted in Létourneau et al.'s (2025) systematic review. Their analysis of 28 ITS studies in K-12 contexts concluded that while most systems yielded positive results, the studies often lacked diversity and rarely explored ethical or fairness dimensions.

Zomer and Frankenberg-Garcia (2021) make an important contribution by demonstrating that fine-tuning language models on L1-influenced learner corpora yields better performance than generic grammatical error correction (GEC) systems. Their work validates the importance of L1-sensitive modeling for writing support, yet it stops short of proposing scalable deployment tools or fairness assessments.

A related concern is scoring bias. Guo et al. (2025) found that automated scoring systems produced “distorted disparities” in evaluating English Language Learners (ELLs), particularly when those learners were underrepresented in the training data. Their results confirm that balanced datasets are essential to mitigate scoring bias and uphold fairness. However, few educational NLP systems incorporate such safeguards proactively.

The current literature provides valuable insights into the capabilities of LLMs, the utility of NLP in education, and the emerging recognition of equity risks. Yet these strands often remain disconnected. Most LLM-based tutoring systems emphasize personalization or fluency but not fairness; most GEC models target accuracy but ignore cultural context; and most multilingual NLP research evaluates system performance but not learner impact.

Chollampatt et al. (2016) adapted a neural language model to support three different first languages (L1s), while Nadejde and Tetreault (2020) modified a general-purpose neural grammatical error correction (GEC) system based on the writer's proficiency level and L1. Both studies focused on student-written corpora.

Takahashi et al. (2020) introduced a method for generating more realistic pseudo-errors by incorporating learners' error tendencies. However, their approach was limited to adjusting the probability of grammatical and spelling mistakes. More recently, Zhou et al. (2019) proposed a novel data synthesis approach that creates error-corrected sentence pairs using two machine translation models of differing quality. Nevertheless, high-quality neural machine translation (NMT) output may not always serve as an adequate reference for GEC tasks.

Although AI has made significant advances in education, key gaps persist across intelligent tutoring systems (ITS), natural language processing (NLP), fairness, and multilingual assessment. ITS research emphasizes pedagogical alignment and context as essential to efficacy (Létourneau et al., 2025), yet most systems underutilize core features such as adaptivity, immediate feedback, and learner modeling across varied settings. Similarly, while Transformer-based NLP models improve error correction, they rarely support metacognitive development or explain corrections in pedagogically meaningful ways. Commercial tools like Grammarly remain closed-source and optimized for native speakers, offering limited adaptability or instructional depth (Zheng and Zhang, 2025).

Fairness remains under-addressed. Despite calls for equitable AI systems (Chinta et al., 2024), few studies implement fairness-aware mechanisms beyond scoring. Multilingual learners are particularly underserved: while some studies explore L1 transfer in writing (Zomer and Frankenberg-Garcia, 2021) or model performance across languages (Gupta et al., 2025), integrated solutions that combine adaptivity, equity, and linguistic diversity are rare.

### 2.7 The present study

Given these findings from existing contributions, there is a critical need to design AI tutors that are explicitly equity aware. Existing ITSs rarely adjust for language background, demographic factors, or cultural knowledge, features that are essential for fair personalization. Building on frameworks like EQUAL AI, fairness-aware machine learning, and educational equity research, the proposed “Equity-Aware AI Tutoring Engine for Multilingual Learners” directly responds to these gaps.

This study addresses these gaps by uniting insights across domains. Unlike conventional ITS models, the proposed system incorporates fairness considerations into its design by monitoring feedback characteristics across L1 and proficiency groups and supporting differentiated learner-oriented feedback (Chinta et al., 2024). Leveraging state-of-the-art NLP Transformer models (Zheng and Zhang, 2025), we generate multi-dimensional feedback beyond grammatical correction, incorporating explanation and scaffolding. In contrast to prior models that treat equity as peripheral, our system embeds fairness at its core through mechanisms such as balanced representation across L1 groups and differentiated scaffolding tailored to learner proficiency.

Our system is also L1-aware, responding to documented gaps in multilingual support. By incorporating L1-specific error patterns identified in prior research (Zomer and Frankenberg-Garcia, 2021) and drawing on fairness-evaluation principles such as those discussed by Guo et al. (2025), the framework uses learner linguistic background as contextual information for feedback generation and subsequent equity-oriented evaluation. For instance, Spanish-influenced syntax is contextualized rather than penalized, fostering inclusion and deeper understanding.

We use simulated GPT-style feedback to avoid proprietary black-box models and enhance interpretability. Deploying via Hugging Face Spaces with real-time interaction and flagging supports transparency and iterative accountability (Holstein et al., 2019).

The study contributes to ongoing efforts to develop transparent and pedagogically aligned alternatives to proprietary educational AI systems (Lan et al., 2024; Zheng and Zhang, 2025), while emphasizing the importance of evaluating accessibility, semantic alignment, and subgroup variation in multilingual contexts. This work bridges disconnected strands in ITS, NLP, fairness, and multilingualism, offering an integrated, equity-oriented tutoring framework aligned with current research needs and ethical imperatives. The system incorporates four equity-oriented components: (1) L1-aware NLP processing that incorporates learners' linguistic backgrounds into feedback generation and evaluation; (2) fairness-oriented diagnostics that examine subgroup differences in feedback accessibility, elaboration, and semantic alignment; (3) culturally responsive and proficiency-sensitive feedback strategies informed by documented L1-specific spelling patterns; and (4) equity monitoring through subgroup-level analyses of feedback length, readability, semantic correspondence, and feedback coverage. These components enable potential disparities to be identified and evaluated without assuming that observed subgroup variation necessarily constitutes algorithmic bias.

Accordingly, the study addresses the following research questions:

1. How do GPT-generated and rule-based simulated feedback differ in linguistic accessibility, feedback elaboration, and semantic alignment for multilingual English learners?
2. To what extent do the length, readability, and semantic alignment of GPT-generated feedback vary across learners' L1 backgrounds and English proficiency levels?
3. To what extent do observed subgroup differences persist after accounting for relevant input characteristics, and what do these patterns suggest about equity in AI-generated feedback?

## 3 Methods

This study employed a multi-phase research design to develop and evaluate an equity-aware AI tutoring system for multilingual English learners, with a focus on personalized spelling support. The methodology integrates corpus linguistics, natural language processing (NLP), fairness-aware model design, and user-centered deployment. The development process was structured to ensure traceability, replicability, and equity throughout all phases, from data acquisition and preprocessing to model training and deployment via an interactive tutoring interface. This approach enabled the creation of a spelling feedback tool that is not only linguistically and culturally responsive but also pedagogically adaptive and technically scalable (Blodgett et al., 2020; García and Kleifgen, 2010).

### 3.1 Dataset acquisition and annotation

This study employed the *TOEFL-Spell corpus* (ETS Corpus of Non-Native Written English), obtained from Linguistic Data Consortium https://catalog.ldc.upenn.edu/LDC2014T06 (Flor et al., 2019), a benchmark dataset containing over 6,000 manually annotated spelling errors extracted from 883 essays written by non-native English speakers. These essays originate from the broader TOEFL11 corpus (Blanchard et al., 2013), LDC2014T06, which comprises 12,100 essays authored by learners from 11 first language (L1) backgrounds, including Arabic, Chinese, French, German, Hindi, Italian, Japanese, Korean, Spanish, Telugu, and Turkish, across three proficiency levels (low, medium, high). The TOEFL11 corpus includes detailed metadata such as L1, writing prompt ID, and proficiency tier, enabling stratified analyses across linguistic and educational subgroups.

Each spelling error in TOEFL-Spell is annotated with its character offsets, the erroneous token, and the corrected form, allowing for sentence-level contextual modeling. Following best practices in educational NLP (Ng et al., 2014), we extracted contextual windows of ±50 characters around each error, replacing the misspelled token with a [MISSPELL] placeholder. The corresponding correction was used as the ground-truth target, enabling compatibility with transformer-based models such as T5 and GECToR (Omelianchuk et al., 2020).

To support fairness-aware evaluation, we merged spelling annotations with TOEFL11 metadata using filename identifiers. This enriched dataset enabled stratified assessments across L1 groups and proficiency levels, aligning with best practices in equitable and interpretable educational AI (Granger et al., 2015; Hashimoto et al., 2018; Rozovskaya and Roth, 2016).

### 3.2 Data preprocessing and context extraction

Each spelling error was mapped to its corresponding essay using character offsets provided in the TOEFL-Spell annotation file. A custom Python script parsed the original essays, replacing the misspelled word with a visually distinct placeholder (e.g., < < hapiness>>) to facilitate both manual inspection and model training. To ensure adequate syntactic and semantic context without revealing the original writing prompt, a five-sentence window surrounding the misspelling was extracted.

To support detailed linguistic profiling, the dataset was augmented with the following derived features:

- Essay segment length (character count),
- Flesch Reading Ease scores for readability, computed using the textstat library (Kincaid et al., 1975),
- Word-level Levenshtein distance to measure edit complexity,
- Error-type classifications (e.g., phonological shifts, transpositions, substitutions), following heuristics adapted from Rozovskaya and Roth (2016).

Misspellings were categorized using rule-based comparison of misspelled and corrected forms, capturing typical learner patterns such as insertions, deletions, and phonological errors, particularly relevant for ELLs from morpho-phonologically distinct L1 backgrounds.

### 3.3 Error highlighting and contextualization

To prepare inputs for LLM-based feedback generation, each spelling error was embedded within its original essay context through inline string replacement (e.g., < hapiness>). This approach visually marked the error while preserving the surrounding sentence structure. A five-sentence window was extracted to replicate authentic writing conditions and ensure pedagogically meaningful feedback. Providing such rich linguistic context aligns with evidence that contextually grounded prompts improve language model reasoning and response quality (Brown et al., 2020).

### 3.4 Simulated feedback generation and prompt design

To emulate personalized feedback without relying on proprietary black-box APIs, this study implemented a rule-based simulation framework designed to replicate the pedagogical structure of GPT-style output. Drawing on prior research into L1-specific orthographic challenges, such as vowel omission among Arabic learners or confusion with silent letters in Chinese L1 users (Cook, 2016), the system produced culturally responsive and linguistically adaptive feedback. The level of elaboration was calibrated according to learner proficiency: beginner learners received simplified guidance, while more advanced learners were provided with metalinguistic explanations. This scaffolding strategy is aligned with Universal Design for Learning (UDL), which emphasizes differentiated instruction to accommodate diverse learner profiles (Meyer et al., 2014).

Each spelling error was transformed into a structured prompt that included the misspelled sentence, the corrected form, and a directive requesting a supportive explanation, memory aid, and encouragement. This design drew on formative feedback principles that advocate for low-stakes, growth-oriented responses emphasizing clarity and motivation (Shute, 2008). For example, a typical prompt was:

“*The learner wrote: ‘hapiness'. The correct spelling is: ‘happiness'. Please explain the mistake gently, offer a memory trick, and encourage the learner.”*

The same general prompting procedure was applied across observations to reduce variation attributable to prompt format. Learner metadata were retained because the study specifically examined whether feedback characteristics varied across linguistic and proficiency groups.

To enable comparative evaluation, two feedback pipelines were implemented. First, 100 prompts were submitted to GPT-4 via the OpenAI API with a tutoring frame and a temperature setting of 0.7 to balance variation and factuality. Feedback was generated once for each of the 32 observations using the same generation protocol. No responses were manually edited or selectively retained on the basis of perceived quality. Second, for comparison, a rule-based simulated feedback engine produced template-driven responses incorporating both L1-specific error patterns and proficiency-sensitive phrasing (e.g., “Arabic speakers may omit vowels”). This hybrid approach allowed for controlled analysis of linguistic quality and pedagogical fairness, in line with emerging best practices in responsible NLP (Bender et al., 2021).

To evaluate equity, the system also included fairness diagnostics: readability metrics (e.g., Flesch-Kincaid scores) and explanation lengths were computed across L1-proficiency subgroups. These indicators were used to flag potential disparities in feedback accessibility or detail, ensuring the system's alignment with inclusive AI design and fairness-aware educational assessment.

### 3.5 Fairness-aware personalization design

To support equitable learning experiences, this study incorporated fairness-aware personalization into the feedback generation process. Drawing on language transfer theory (Odlin, 2003), feedback messages were tailored to address common L1-specific spelling challenges. For instance, Spanish-speaking learners frequently insert vowels before /s/-clusters (e.g., “eschool”), while Arabic speakers often omit short vowels. These patterns were embedded into feedback heuristics to increase cultural and linguistic relevance. Additionally, the tone, length, and complexity of explanations were calibrated according to learners' English proficiency levels. Simpler messages and encouragement were prioritized for lower-proficiency users, while metalinguistic detail was added for advanced learners. This strategy aligns with Vygotsky and Cole's (1978) scaffolding theory, which emphasizes differentiated instruction based on learners' developmental stages.

### 3.6 Equity-centered feedback evaluation

To evaluate the equity and cognitive accessibility of system-generated feedback, a suite of quantitative metrics was computed across first-language (L1) and proficiency subgroups. Feedback length, measured in character count, served as an indicator of elaboration and richness. Readability was assessed using the Flesch Reading Ease score (Kincaid et al., 1975), implemented via the textstat Python library. In addition, subgroup coverage was calculated as the proportion of learners who received valid feedback outputs. Subgroups with significantly lower readability or length were flagged for potential equity risks, consistent with recent frameworks for fairness in educational NLP (Baker and Hawn, 2022). Particular attention was paid to learners from L1s with known orthographic and phonological divergence from English, such as Arabic and Chinese, to ensure inclusivity in system performance (Laufer and Girsai, 2008).

As shown in Figure 1, the fairness scoring module evaluates discrepancies in feedback length and readability across L1 group.

**Figure 1:**
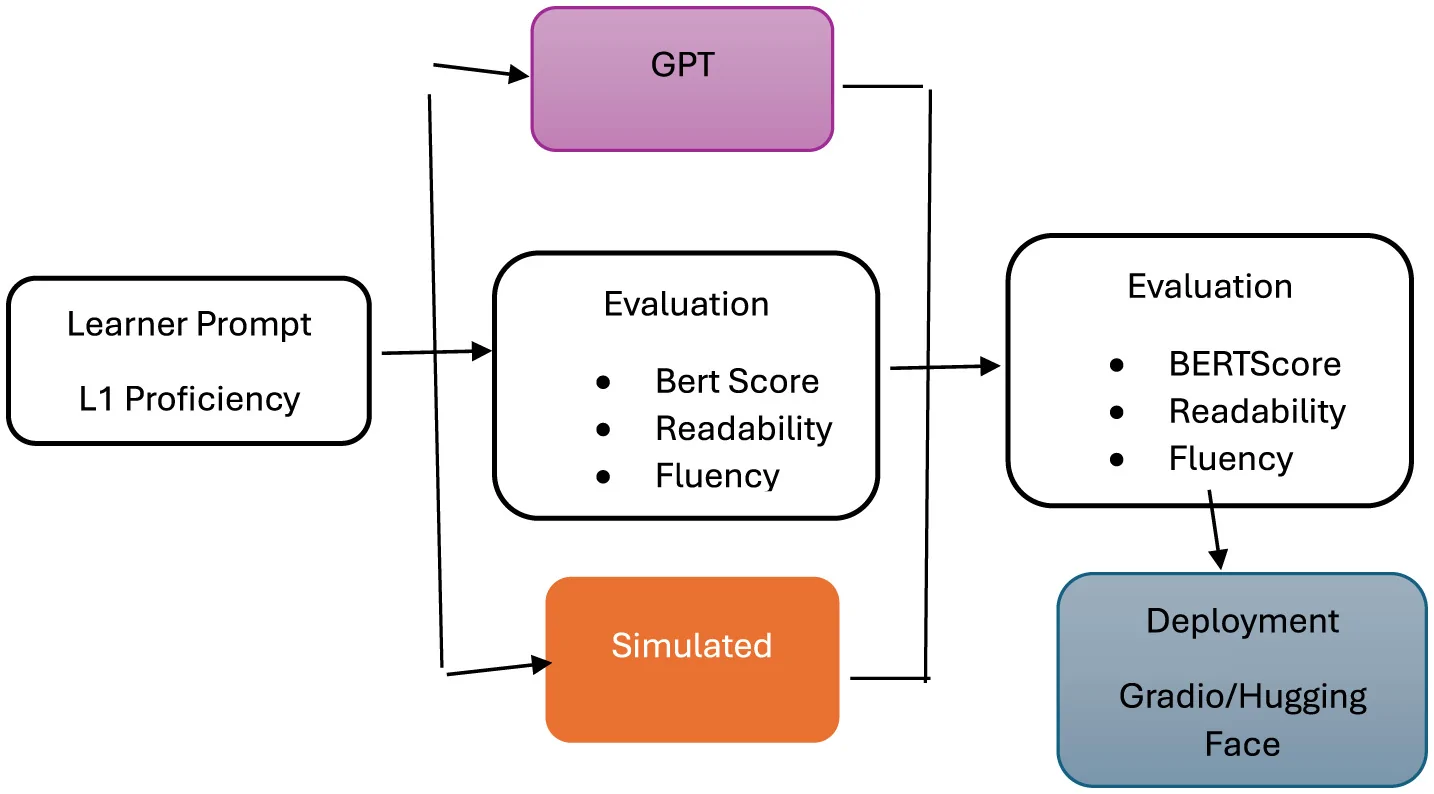
Conceptual architecture of the proposed fairness-aware AI tutoring framework, integrating GPT-generated feedback, rule-based scaffolding, and fairness assessment tools.

### 3.7 Visualization and error pattern profiling

To support interpretability and model validation, we visualized several patterns within the annotated dataset. Bar charts and tabular summaries illustrated: (1) the most frequent misspellings by L1, (2) distributions of error types across proficiency levels, (3) the average readability and length of GPT-generated vs. simulated feedback, and (4) subgroup-level feedback coverage. These analyses confirmed the presence of well-documented L1-specific spelling issues, such as vowel omissions in Arabic or phonologically approximated English spellings in Chinese, consistent with prior learner corpus research (Granger et al., 2015).

### 3.8 Comparative analysis of feedback generation approaches

To explore trade-offs between accuracy, adaptability, and fairness, we compared GPT-generated feedback to rule-based simulated responses. Simulated feedback, constructed using template-based heuristics, ensured consistency and transparency, particularly under resource constraints. Meanwhile, GPT-4 responses offered greater expressiveness, fluency, and personalization, dynamically adjusting to input context and learner attributes. Both approaches were evaluated across L1 and proficiency strata, with metrics such as lexical richness, tone, and scaffolding depth. This dual-path strategy allowed us to assess pedagogical efficacy and inclusivity, offering insights into the design of equitable AI tutors capable of supporting linguistically diverse learners.

### 3.9 Fine-tuning dataset preparation

To facilitate future supervised learning and model refinement, we constructed a fine-tuning dataset comprising over 1,000 prompt-response pairs. Each entry was saved in a two-column format with input_text (the instructional prompt) and output_text (the personalized feedback), following conventions used in instruction-tuned architectures such as T5, FLAN-T5, and GPT-J (Raffel et al., 2020). Prompts included learner-specific context, such as L1 background and proficiency level, embedded within the text to support domain adaptation. This dataset, saved as finetune_dataset.csv as shown in the Appendix, enables reproducible training of models focused on culturally and linguistically responsive spelling feedback.

### 3.10 Evaluation metrics

To assess feedback characteristics and semantic correspondence, the study employed complementary quantitative evaluation measures. The broader system-development evaluation considered GPT-generated, static simulated, and adaptive simulated feedback across several technical and pedagogical dimensions. For the verified matched analysis reported in the statistical evaluation, however, semantic similarity was assessed specifically between GPT-generated feedback and the corresponding rule-based simulated feedback for the 32 complete paired observations.

BERTScore was used because it evaluates contextual semantic correspondence beyond surface-level lexical overlap through transformer-based contextual embeddings (Zhang et al., 2019). Using the bert_score Python library, precision, recall, and F1 were computed for each matched GPT-simulated feedback pair. Precision represents the degree to which semantic content in the generated response corresponds to the comparison text, recall reflects the extent to which semantic information in the comparison text is represented in the generated response, and F1 summarizes their balance.

In the analysis, BERTScore was therefore interpreted as a measure of semantic correspondence between GPT-generated and simulated feedback rather than as direct evidence of pedagogical accuracy or instructional effectiveness. This distinction is important because the simulated feedback constituted a computational comparison response rather than an independently human-rated gold-standard explanation. BERTScore F1 was additionally examined across L1 and proficiency subgroups to determine whether semantic correspondence varied systematically across the learner groups represented in the analytical sample.

### 3.11 Statistical analysis and fairness evaluation

Descriptive statistics were computed for GPT-generated and simulated feedback across first-language (L1) and proficiency groups, including sample size, mean, standard deviation, median, and 95% confidence intervals. Because subgroup sizes were unequal and several outcome distributions departed from normality, between-group differences were assessed using Kruskal-Wallis tests, with epsilon-squared reported as an effect-size measure. Where omnibus differences were significant, pairwise Mann-Whitney U-tests with Holm correction were used to control for multiple comparisons, and rank-biserial correlations were reported as effect sizes. Paired GPT-vs.-simulated comparisons were evaluated using Wilcoxon signed-rank tests with paired rank-biserial effect sizes. Semantic similarity between GPT-generated and simulated feedback was assessed using BERTScore precision, recall, and F1. Exploratory robust regression models with HC3 standard errors were additionally used to examine whether observed differences persisted after accounting for prompt length, misspelling length, and spelling-length difference. Fairness was operationalized as systematic variation in feedback quality and accessibility across learner groups after considering relevant learner- and input-level characteristics; consequently, descriptive subgroup differences were treated as disparities rather than evidence of algorithmic bias unless supported by inferential analysis. Because L1 and proficiency were not fully crossed in the sample, their independent effects could not be completely disentangled.

### 3.12 System deployment and reproducibility

The tutoring framework was implemented as a Gradio-based interactive interface and deployed through Hugging Face Spaces. The interface enables a user to enter a misspelled word and its intended correction, specify L1 background and proficiency level, and receive learner-oriented feedback. The deployment served as a proof-of-concept implementation of the proposed framework rather than as a formal evaluation of learner outcomes or usability.

Analysis scripts, generated feedback, intermediate datasets, and processed outputs were retained to support reproducibility and methodological auditing. The verified final analytical dataset contained 32 one-to-one matched GPT and simulated feedback observations with no duplicate analytical records or missing feedback outcomes. No claims regarding learning gains, usability effectiveness, or behavioral change were made because these outcomes were not formally evaluated in the present study.

### 3.13 Ethical considerations and reproducibility

To ensure transparency and ethical compliance, this study followed established best practices for AI research in education (Mitchell et al., 2019). All models, outputs, and feedback logs were archived to support future audits and reproducibility. Simulated feedback was explicitly labeled to distinguish it from generative model outputs and avoid misleading hallucinations. The full development pipeline was modularized and released as an open-source package (equity_ai_tutor.zip), including core scripts (app.py, requirements.txt) and processed outputs (pipeline_output_full.csv). User inputs in the deployed interface were anonymized, and no essay content was retained post-inference, safeguarding learner privacy and data ethics. The study relied on secondary, anonymized learner-corpus data and did not involve direct interaction with research participants during the computational evaluation. No identifiable personal information was collected as part of the analysis. The interactive deployment was designed to process learner inputs for feedback generation without requiring personally identifying information. Ethical interpretation of model behavior additionally followed a conservative fairness framework in which observed group differences were not equated with algorithmic discrimination without supporting statistical evidence.

## 4 Results

The development and evaluation of the Equity-Aware AI Tutoring Engine produced empirical findings across linguistic, technical, pedagogical, and equity-related dimensions. These findings arose from complementary stages of analysis involving corpus annotation, contextualized feedback generation, rule-based feedback simulation, GPT-generated feedback, fairness diagnostics, semantic evaluation, and interactive system deployment. More than 6,000 annotated spelling instances from the TOEFL-Spell resource were used in the corpus-level and simulation stages. A smaller matched evaluation sample was used for direct comparison of GPT-generated and simulated feedback. Following completion and verification of previously missing GPT outputs, this comparative sample comprised 32 observations with complete GPT and simulated responses and no duplicate analytical records. The distinction between the corpus-level analyses and the matched feedback-evaluation sample is maintained throughout the results to avoid conflating descriptive corpus patterns with model-level comparisons.

### 4.1 Spelling error distribution across L1 groups

From the TOEFL-Spell subset, a total of 6,121 annotated spelling errors were extracted, of which approximately 91.4% were classified as type “M” (nonword misspellings), consistent with previous learner-corpus research identifying nonword forms as a prominent category of spelling error among English learners (Flor et al., 2019). Linking the spelling annotations to TOEFL11 metadata enabled examination of error patterns across learners' first-language backgrounds.

Descriptive variation was observed across L1 groups, with recurrent forms including *theorical, pratical*, and *unefficient* among Japanese learners, *bussiness* and *succesful* among Chinese learners, and *infomation* among Turkish learners (Table 1). Such patterns are compatible with research suggesting that L2 spelling development can be influenced by cross-linguistic differences in orthographic and phonological systems (Koda, 2005; Cook, 1997). They are also broadly consistent with contrastive interlanguage perspectives emphasizing the potential contribution of cross-linguistic transfer to learner-language patterns (Granger et al., 2015; Cook, 2016).

**Table 1:** Top 5 misspellings per L1 (first language).

L1	Misspelling	Count
Arabic	beacuse	1
	enviroment	1
	writi	1
Chinese	bussiness	2
	comfront	1
	escencial	1
	peaople	1
	succesful	1
Japanese	Theorical	4
	pratical	2
	unefficient	2
	attractiv	1
	futur	1
Spanish	Ofcourse	1
	insited	1
	knowledege	1
	paediatrist	1
	wihin	1
Turkish	infomation	2
	interesing	1
	loger	1
	penonmenon	1
	wnat	1

Nevertheless, these descriptive patterns should not be interpreted as demonstrating that membership in a particular L1 group causes greater spelling difficulty. Differences in subgroup representation, learner proficiency, lexical characteristics, and error composition provide plausible alternative explanations and are considered in the subsequent fairness analysis.

### 4.2 Distribution across first-language groups

Within the matched feedback-evaluation sample, Japanese learners represented the largest L1 subgroup (*n* = 11), followed by Chinese (*n* = 7), Turkish (*n* = 6), Spanish (*n* = 5), and Arabic learners (*n* = 3) (Figure 2). These counts describe the composition of the analytical sample rather than population-level prevalence of spelling errors.

**Figure 2:**
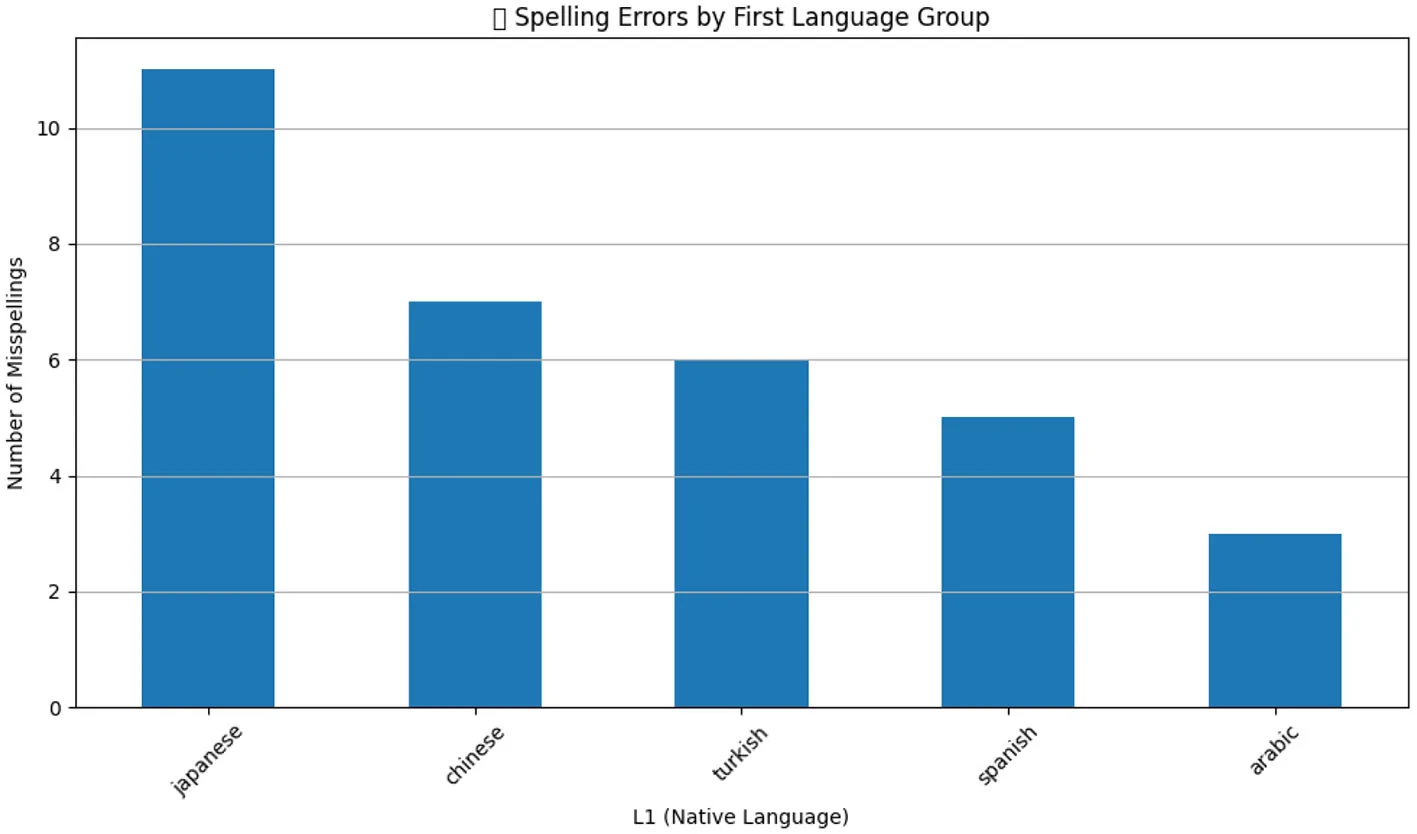
Distribution of observations (spelling error) by first-language group.

The greater representation of some L1 groups is relevant to interpretation of subsequent comparisons because unequal subgroup sizes affect statistical precision. Although previous work has demonstrated that orthographic distance and language-transfer processes may influence second-language spelling (Koda, 2005; Swan and Smith, 2001), the present subgroup frequencies cannot independently establish such effects.

### 4.3 Spelling errors by proficiency level

The distribution (Figure 3) shows that low-proficiency learners made the highest number of spelling errors (*n* = 14), followed by mid-proficiency learners (*n* = 13), while high-proficiency learners made substantially fewer errors (*n* = 5). This gradient indicates a general decline in spelling errors as proficiency increases. The descriptive distribution therefore contained greater representation of low- and mid-proficiency learners than high-proficiency learners. This pattern is consistent with second language acquisition theory, where orthographic and lexical control typically develop with increasing proficiency. The elevated error rates among lower-proficiency learners highlight the importance of tailored feedback that addresses both surface-level accuracy and deeper linguistic scaffolding (Hyland and Hyland, 2006). Furthermore, the persistence of errors among mid-level learners suggests that targeted support during the “interlanguage plateau” stage remains crucial (Selinker, 2015). Second-language acquisition research indicates that orthographic and lexical control generally develops alongside language proficiency and that appropriately scaffolded feedback can be particularly important during developing stages of language learning (Hyland and Hyland, 2006; Selinker, 2015). However, the frequencies shown here describe sample composition and should not themselves be interpreted as evidence that low-proficiency learners have a statistically greater error rate.

**Figure 3:**
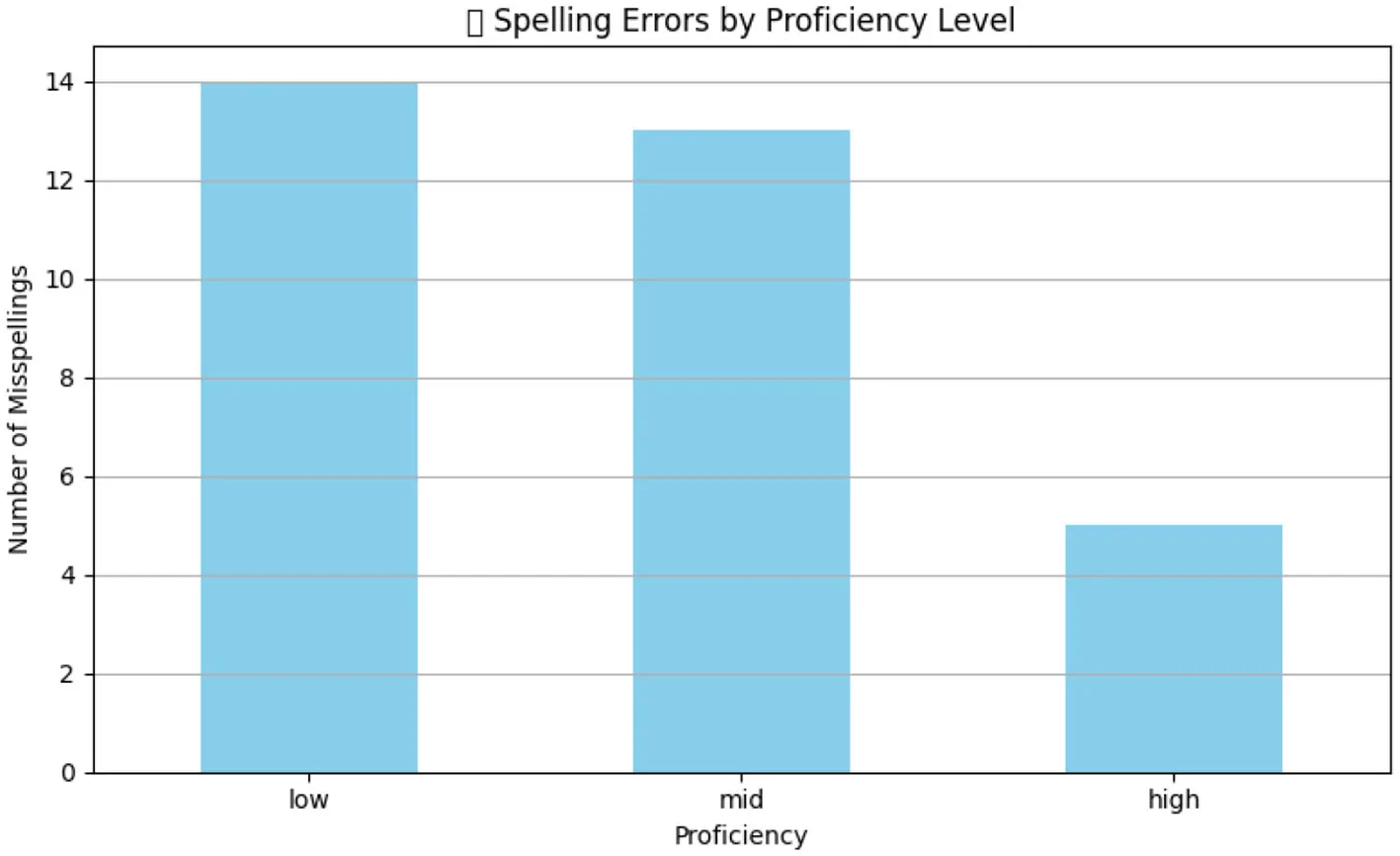
Distribution of observations by proficiency level.

### 4.4 Average GPT explanation length by proficiency

Following completion of all previously missing GPT outputs, the matched analysis included valid GPT feedback for all 32 observations. GPT-generated responses averaged 657.43 (Figure 4) characters for low-proficiency learners (SD = 119.93), 557.39 characters for mid-proficiency learners (SD = 136.74), and 602.00 characters for high-proficiency learners (SD = 145.64).

**Figure 4:**
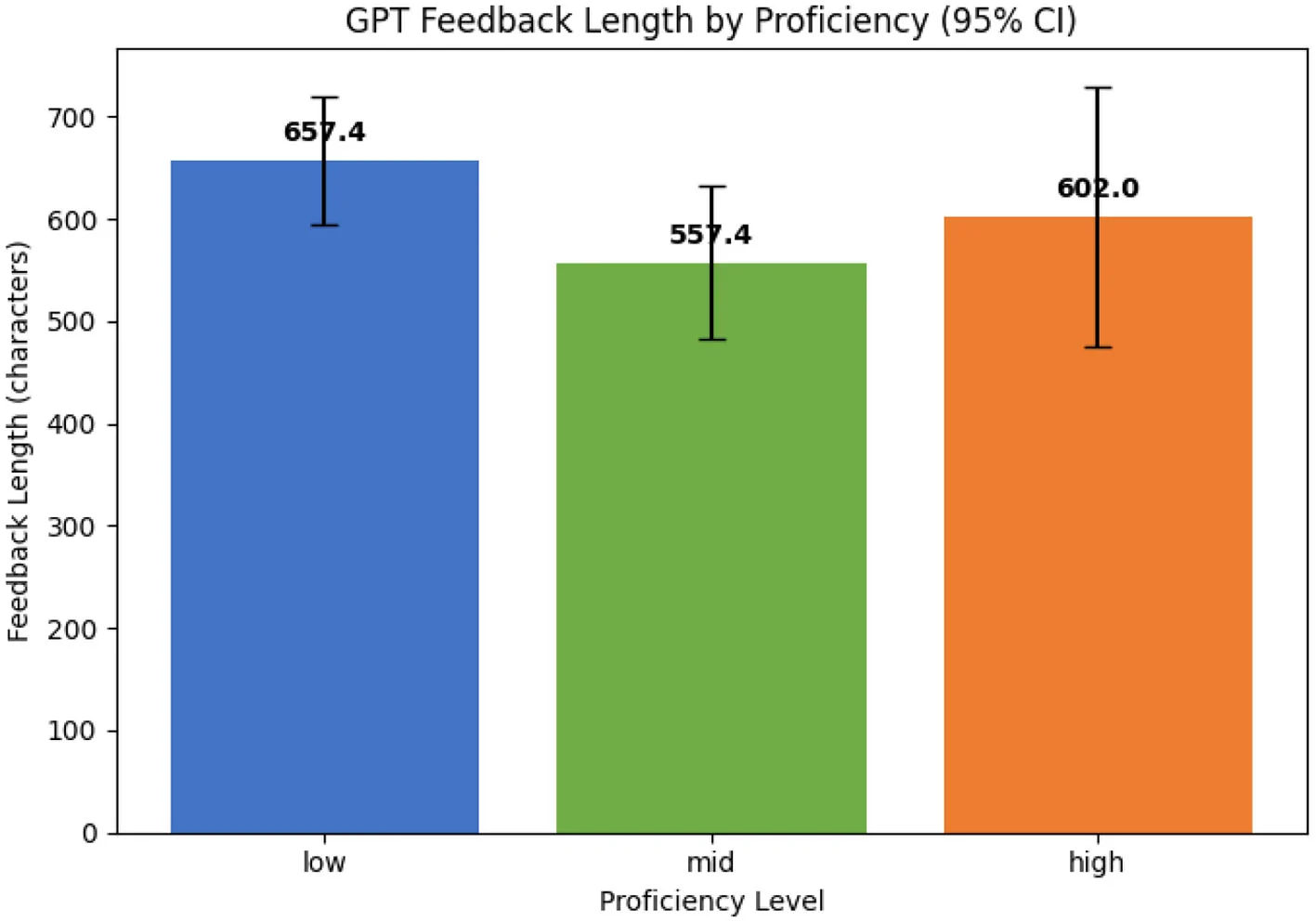
Mean GPT-generated feedback length by proficiency level.

Although the descriptive means indicate variation in response length, the Kruskal-Wallis test showed that these differences were not statistically significant, H(2) = 1.724, *p* = 0.422, ε^2^ = 0.000. Accordingly, the completed analysis does not provide evidence that GPT systematically altered the amount of feedback according to proficiency level.

This finding qualifies the preliminary interpretation that longer feedback for particular proficiency groups necessarily represented adaptive scaffolding. Although differentiated feedback is theoretically consistent with adaptive tutoring and scaffolding principles that intelligent systems offer greater scaffolding at intermediate stages (Graesser et al., 2005; Shute, 2008), response length alone is insufficient to establish pedagogically appropriate personalization.

### 4.5 Readability of GPT feedback across L1 groups

The completed analysis showed that GPT-generated feedback remained reasonably readable across all five represented L1 groups. Mean Flesch Reading Ease scores were 78.92 (Figure 5) for Turkish learners (SD = 6.11), 78.72 for Chinese learners (SD = 4.02), 78.04 for Arabic learners (SD = 6.99), 73.39 for Spanish learners (SD = 8.38), and 72.22 for Japanese learners (SD = 6.17).

**Figure 5:**
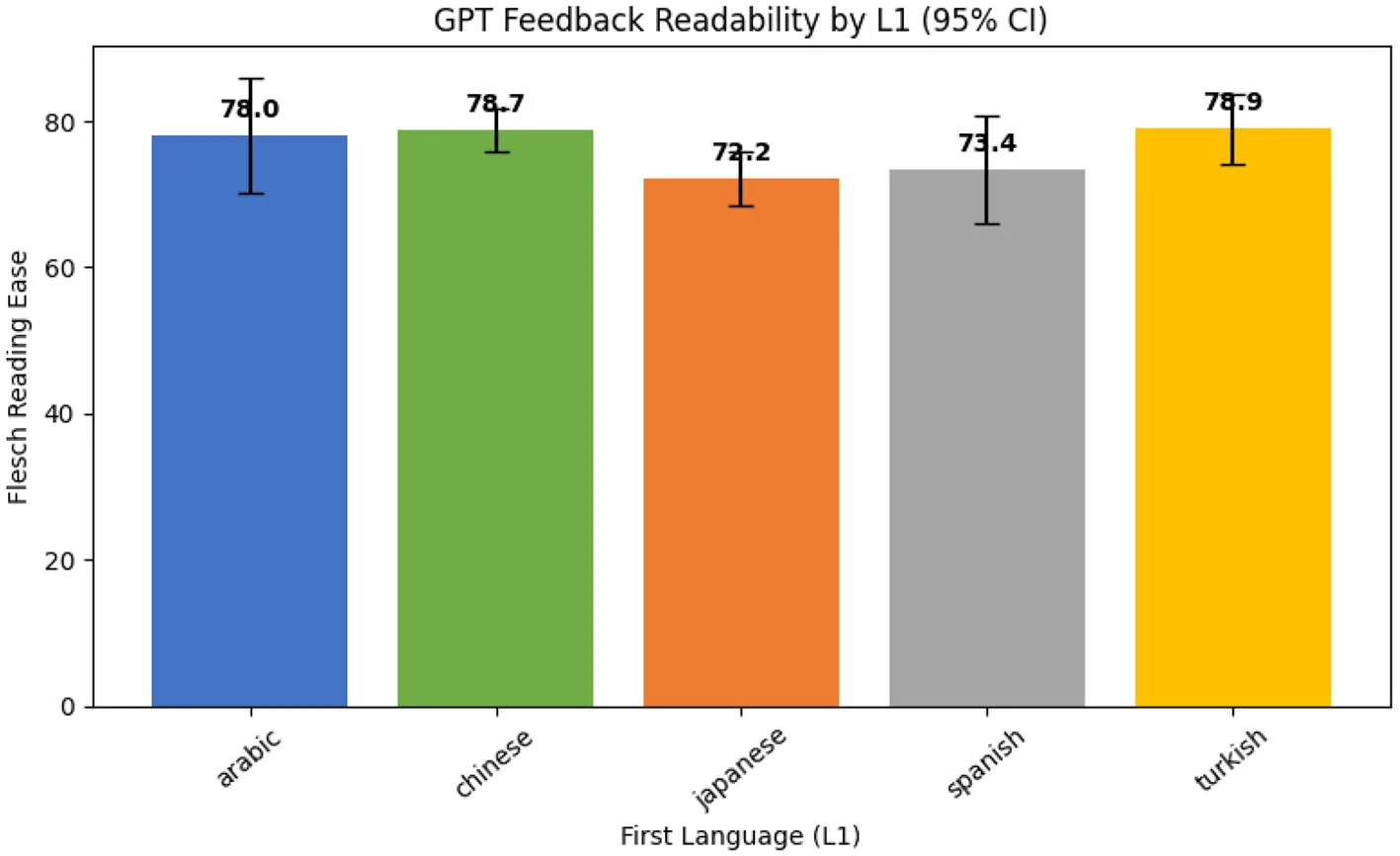
Mean GPT feedback readability across L1 groups with 95% confidence intervals.

Although some descriptive variation was evident, the difference across L1 groups was not statistically significant, H(4) = 7.547, *p* = 0.110, ε^2^ = 0.131. Thus, the corrected analysis does not support the earlier conclusion that GPT feedback accessibility differed systematically by L1.

This distinction is particularly important for the fairness interpretation. Readability is relevant to linguistic accessibility for multilingual learners (Crossley et al., 2008), but variation in readability alone does not constitute evidence of algorithmic or sociolinguistic bias. The results therefore indicate descriptive heterogeneity rather than a statistically established L1-based accessibility disparity.

### 4.6 Distribution of GPT feedback across L1-proficiency groups

All 32 observations in the final matched dataset contained valid GPT-generated feedback. The sample consisted of Arabic low-proficiency (*n* = 3), Japanese low-proficiency (*n* = 11), Chinese mid-proficiency (*n* = 7), Turkish mid-proficiency (*n* = 6), and Spanish high-proficiency (*n* = 5) observations (see Figure 6).

**Figure 6:**
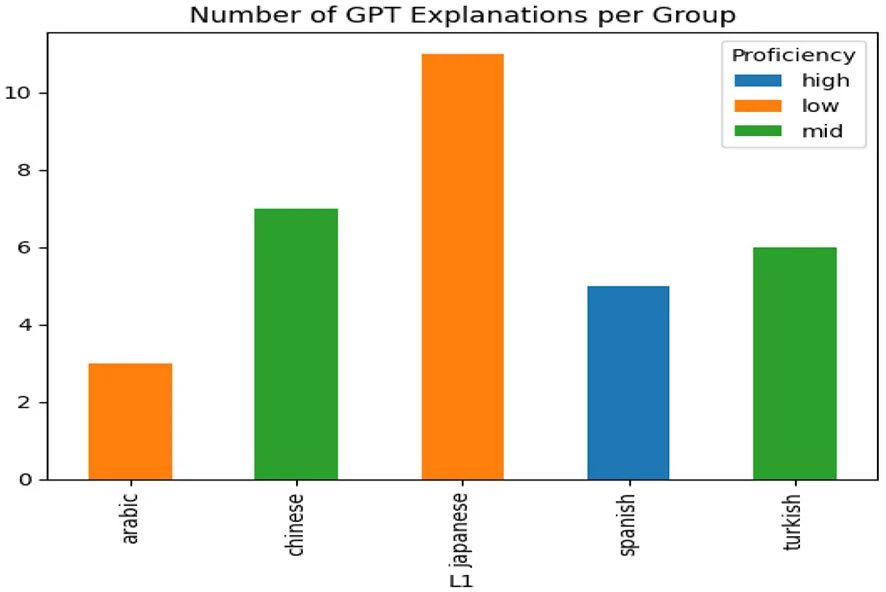
Distribution of the matched evaluation sample by L1 and proficiency.

This distribution identifies an important limitation in the study design: L1 and proficiency were not fully crossed. In this analytical sample, particular L1 groups occurred only within particular proficiency categories. Consequently, descriptive differences attributed to L1 may partly reflect proficiency composition, while apparent proficiency differences may partly reflect L1 composition. This confounding is explicitly considered in the interpretation of the fairness analyses.

### 4.7 GPT vs. simulated feedback: readability and length

GPT-generated feedback was substantially more elaborated than rule-based simulated feedback. Across the 32 paired observations, GPT responses averaged 608.12 characters, compared with 218.69 characters for simulated responses (see Figure 7a). A Wilcoxon signed-rank test confirmed a statistically significant paired difference, *W* = 0.00, *p* < 0.001, with a paired rank-biserial correlation of *r* = 1.00, indicating a very large difference in feedback length.

**Figure 7:**
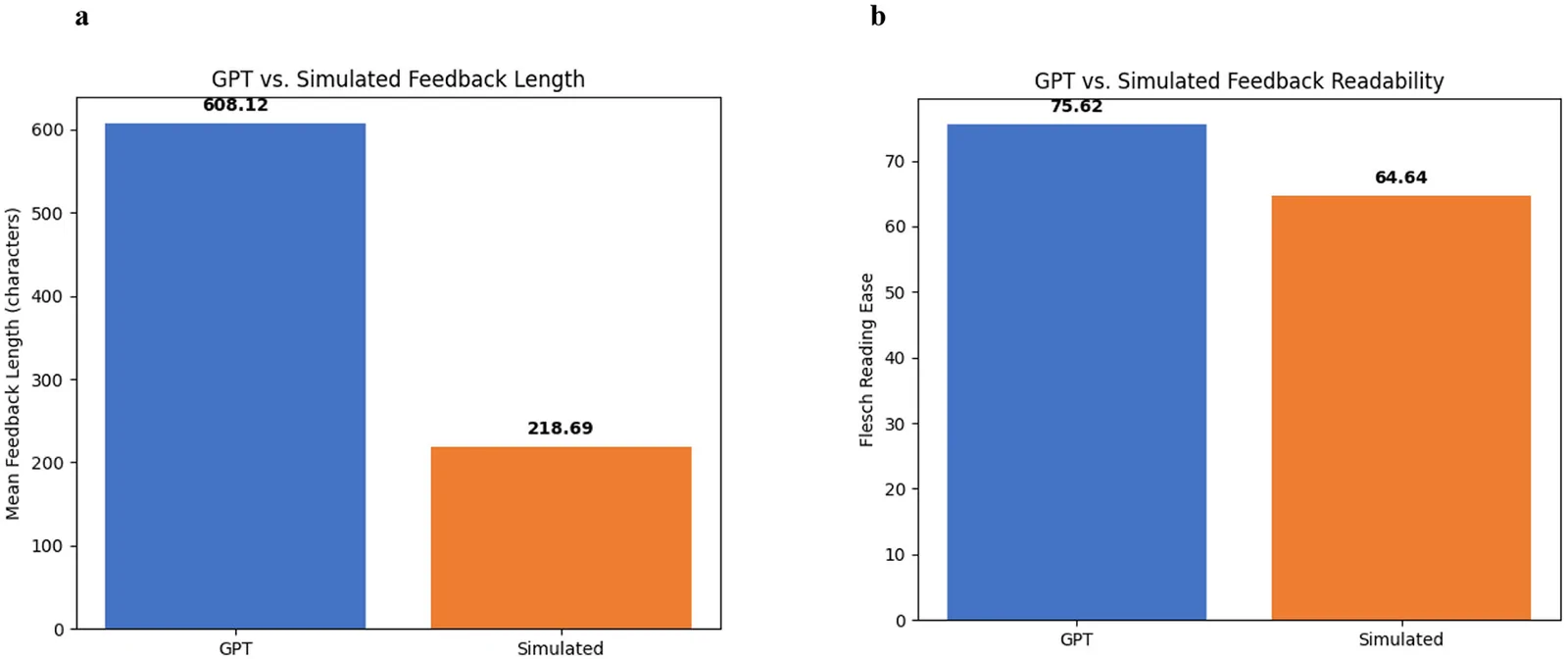
**(a, b)** Average feedback length: GPT vs. simulated.

The completed analysis also changed the preliminary interpretation of readability. GPT feedback had a mean Flesch Reading Ease score of 75.62, compared with 64.64 for simulated feedback (see Figure 7b). This difference was statistically significant, *W* = 22.00, *p* < 0.001, with a large paired rank-biserial effect size (*r* = 0.917). Thus, in the verified sample, GPT responses were both longer and more readable on average than simulated responses.

These findings suggest that greater elaboration did not necessarily impose greater textual complexity. This is pedagogically relevant because detailed feedback can provide explanatory scaffolding, while accessibility remains important for learners with developing English proficiency (Shute, 2008; Wang and Vásquez, 2012). Nevertheless, Flesch Reading Ease measures textual characteristics rather than actual learner comprehension, and the results should therefore not be interpreted as direct evidence that GPT feedback produced superior learning outcomes.

### 4.8 Feedback personalization by learner proficiency

GPT feedback exhibited descriptive variation across proficiency groups, but the inferential findings provide limited support for systematic proficiency-sensitive personalization. As reported above, feedback length did not differ significantly by proficiency, H(2) = 1.724, *p* = 0.422, ε^2^ ~0.000.

For readability, mid-proficiency learners received the highest mean Flesch Reading Ease score (M = 78.81, SD = 4.86), compared with low-proficiency (M = 73.47, SD = 6.55) and high-proficiency learners (M = 73.39, SD = 8.38). The omnibus Kruskal-Wallis test reached statistical significance, H(2) = 6.125, *p* = 0.047, ε^2^ = 0.142.

However, Holm-adjusted pairwise tests showed that no individual proficiency comparison remained statistically significant after correction for multiple testing. The low-vs.-mid comparison produced *U* = 44.00, unadjusted *p* = 0.024, rank-biserial *r* = 0.517, but Holm-adjusted *p* = 0.072. The low-vs.-high comparison was non-significant (pHolm = 0.964), as was the mid-vs.-high comparison (pHolm = 0.189).

Accordingly, the results provide preliminary evidence of variation in feedback accessibility but do not demonstrate robust proficiency-specific adaptation. This more cautious interpretation is consistent with scaffolding theory, which emphasizes matching instructional assistance to learner needs (Shute, 2008; Vygotsky and Cole, 1978), while recognizing that length and readability are indirect rather than definitive indicators of pedagogical personalization.

### 4.9 Readability and fairness across L1 group

The completed GPT responses showed relatively comparable readability across the represented L1 groups. Arabic (M = 78.04), Chinese (M = 78.72), and Turkish (M = 78.92) feedback had somewhat higher mean Flesch scores than Spanish (M = 73.39) and Japanese feedback (M = 72.22) (Figure 8). However, the overall L1 difference was not statistically significant, H(4) = 7.547, *p* = 0.110, ε^2^ = 0.131.

**Figure 8:**
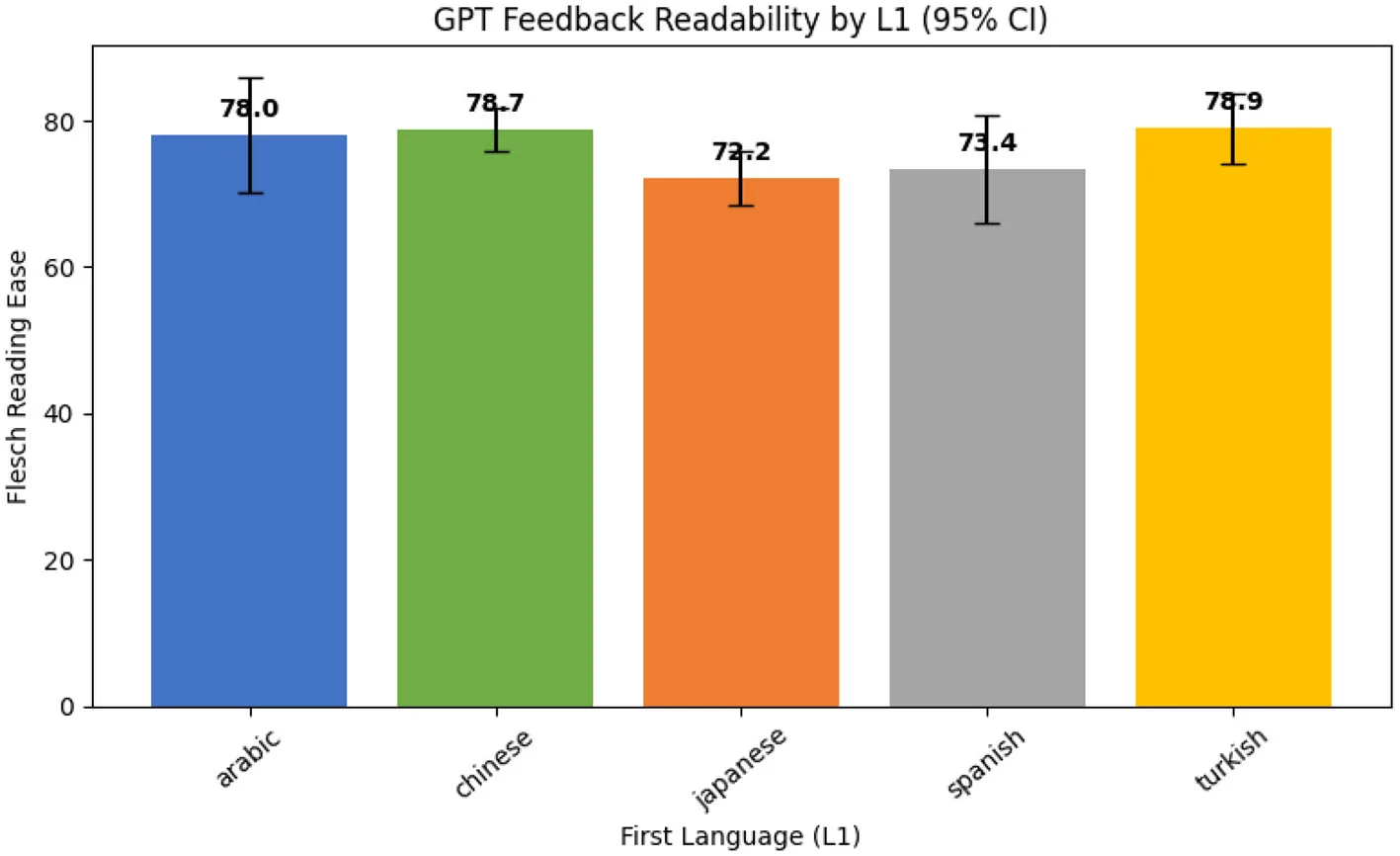
GPT feedback readability by L1 with 95% confidence intervals.

Similarly, GPT feedback length varied descriptively across L1 groups, Arabic M = 543.00, Chinese M = 591.14, Japanese M = 688.64, Spanish M = 602.00, and Turkish M = 518.00, but the omnibus test was non-significant, H(4) = 6.862, *p* = 0.143, ε^2^ = 0.106.

These findings differ materially from the preliminary analysis in which missing GPT responses produced zero length and readability values for several groups. After completing the missing responses and rerunning the analysis, the data do not provide sufficient evidence that GPT systematically disadvantaged particular L1 groups in either feedback length or readability.

This does not establish that the model is “fair.” Rather, it means that this study did not detect statistically supported L1 disparities on these specific indicators. Algorithmic fairness is multidimensional, and observed differences may arise from error characteristics, learner proficiency, input composition, sampling, or other contextual factors (Blodgett et al., 2020; Sap et al., 2021).

### 4.10 Simulated feedback readability and length

Rule-based simulated feedback also exhibited variation across L1 groups. Mean readability was highest for Spanish learners (M = 71.41), followed by Japanese (M = 66.25), Turkish (M = 62.57), Chinese (M = 61.73), and Arabic learners (M = 58.46). Unlike the GPT readability results, this L1 difference was statistically significant, H(4) = 22.294, *p* < 0.001, ε^2^ = 0.678.

Simulated feedback length likewise differed significantly across L1 groups, H(4) = 26.046, *p* < 0.001, ε^2^ = 0.817, and across proficiency groups, H(2) = 24.955, *p* < 0.001, ε^2^ = 0.792. Simulated readability also varied significantly by proficiency, H(2) = 15.208, *p* < 0.001, ε^2^ = 0.455.

These results demonstrate that deterministic or template-driven feedback does not necessarily produce identical linguistic characteristics across learner groups. Because the simulated system explicitly incorporated L1- and proficiency-sensitive rules, however, these differences should be interpreted primarily as consequences of the personalization design rather than as evidence of emergent algorithmic bias. The findings reinforce the need to evaluate linguistic accessibility even in transparent rule-based educational systems (Crossley et al., 2008; Cummins, 2000).

### 4.11 Feedback sample

A qualitative inspection of paired feedback examples further illustrates the distinction between the two feedback approaches. GPT-generated responses generally provided more elaborated explanations, encouragement, and contextual information, whereas simulated responses were more concise and structurally constrained. This qualitative pattern is consistent with the quantitative analysis showing substantially greater average GPT feedback length. However, individual examples should be interpreted illustratively rather than as evidence of group-level differences (VanLehn, 2011).

### 4.12 Personalized feedback simulation

Rule-based personalized feedback was generated across more than 6,000 annotated spelling instances using templates incorporating error characteristics, learner L1, and proficiency information. Feedback messages were evaluated for length and readability using the Flesch Reading Ease measure (Kincaid et al., 1975). The simulation stage was designed to examine the feasibility of differentiated instructional scaffolding at corpus scale rather than to provide a direct statistical comparison with the smaller GPT evaluation sample.

Variation in simulated feedback characteristics reflected the explicit personalization rules incorporated into the templates. This design was informed by Universal Design for Learning principles emphasizing differentiated instructional support according to learner needs (Meyer et al., 2014).

### 4.13 Fairness evaluation across linguistic backgrounds

Fairness was operationalized as the examination of systematic variation in feedback accessibility, elaboration, and semantic alignment across learner groups, while considering plausible learner- and input-level explanations. Feedback length was used as an indicator of elaboration, Flesch Reading Ease as an indicator of linguistic accessibility, and BERTScore F1 as an indicator of semantic correspondence. These variables were treated as fairness-relevant diagnostic measures rather than direct measures of algorithmic bias, consistent with recommendations that disparities in NLP outcomes should be interpreted in relation to their social, linguistic, and data-generating context (Blodgett et al., 2020).

For GPT feedback, neither length, H(4) = 6.862, *p* = 0.143, ε^2^ = 0.106, nor readability, H(4) = 7.547, *p* = 0.110, ε^2^ = 0.131, differed significantly across L1 groups. BERTScore F1 was similarly stable across L1 groups, H(4) = 1.789, *p* = 0.775, ε^2^ ~ 0.000. BERTScore means ranged narrowly from 0.853 to 0.858 across the five L1 groups.

These findings provide no statistically supported evidence of systematic L1 disparities on the three measures examined. However, absence of significant differences should not be equated with proof of algorithmic fairness. The small and uneven subgroup sizes, non-crossed L1-proficiency structure, and limited set of fairness indicators constrain the strength of conclusions that can be drawn.

### 4.14 Statistical analysis of subgroup differences

As summarized in Table 2, the inferential analyses provided little evidence of systematic subgroup differences in GPT-generated feedback. Neither GPT feedback length nor BERTScore F1 differed significantly across L1 or proficiency categories, and GPT readability did not differ significantly across L1. The omnibus test for GPT readability across proficiency groups reached the conventional significance threshold (*p* = 0.0468); however, none of the corresponding pairwise comparisons remained significant following Holm correction. In contrast, simulated feedback exhibited significant L1- and proficiency-related variation in both length and readability, with moderate-to-large epsilon-squared effect sizes.

**Table 2:** Inferential tests of feedback differences across L1 and proficiency groups.

Outcome	Grouping variable	*H*	*p*	*ε^2^*
GPT length	L1	6.862	0.1434	0.106
GPT length	Proficiency	1.724	0.4223	0.000
GPT readability	L1	7.547	0.1097	0.131
GPT readability	Proficiency	6.125	**0.0468**	0.142
Simulated length	L1	26.046	**< 0.001**	0.817
Simulated length	Proficiency	24.955	**< 0.001**	0.792
Simulated readability	L1	22.294	**< 0.001**	0.678
Simulated readability	Proficiency	15.208	**< 0.001**	0.455
BERTScore F1	L1	1.789	0.7745	0.000
BERTScore F1	Proficiency	1.388	0.4996	0.000

H = Kruskal-Wallis statistic; ε^2^ = epsilon-squared effect size. Significant *p*-values are shown in bold. The significant omnibus proficiency effect for GPT readability did not produce a statistically significant pairwise comparison after Holm correction.

### 4.15 Input-adjusted sensitivity analysis

To examine whether apparent subgroup differences could be associated with input characteristics, exploratory regression analyses were conducted using HC3 heteroscedasticity-robust standard errors and controlling for prompt length, misspelling length, and spelling-length difference. The overall L1-adjusted model for GPT feedback length was not statistically significant (*p* = 0.075), nor was the corresponding proficiency-adjusted model (*p* = 0.129). For GPT readability, the L1-adjusted model approached but did not reach statistical significance (*p* = 0.058). Although the overall proficiency-adjusted readability model was significant (*p* = 0.021), neither the low- nor mid-proficiency coefficient independently reached significance after adjustment.

Likewise, neither the L1-adjusted (*p* = 0.181) nor proficiency-adjusted (*p* = 0.136) BERTScore F1 model was statistically significant overall. These analyses provide limited evidence that learner-group membership independently accounted for the observed GPT feedback characteristics after the measured input features were considered.

However, the models are interpreted as exploratory because L1 and proficiency were not fully crossed within the evaluation sample. Arabic and Japanese observations occurred within the low-proficiency category, Chinese and Turkish within the mid-proficiency category, and Spanish within the high-proficiency category. Consequently, the independent effects of L1 and proficiency cannot be completely disentangled. In addition, the relatively small sample size limits statistical power and the stability of multivariable estimates.

### 4.16 Semantic alignment between GPT and simulated feedback

Semantic correspondence between GPT-generated and rule-based simulated feedback was evaluated using BERTScore precision, recall, and F1. Across the 32 matched feedback pairs, the overall BERTScore precision was 0.8370, recall was 0.8742, and F1 was 0.8552, indicating a relatively high degree of semantic correspondence between the two feedback approaches. The higher recall relative to precision suggests that GPT-generated feedback captured much of the semantic content represented in the simulated feedback while also providing additional wording or explanatory content that was not directly matched by the comparison text.

Semantic alignment was highly consistent across the five represented L1 groups. Mean BERTScore F1 values were 0.854 for Arabic, 0.856 for Chinese, 0.853 for Japanese, 0.858 for Spanish, and 0.855 for Turkish learners (Figure 9). Although Spanish learners showed the highest descriptive mean and Japanese learners the lowest, the absolute differences were small and were not statistically significant, H(4) = 1.789, *p* = 0.775, ε^2^ ~ 0.000.

**Figure 9:**
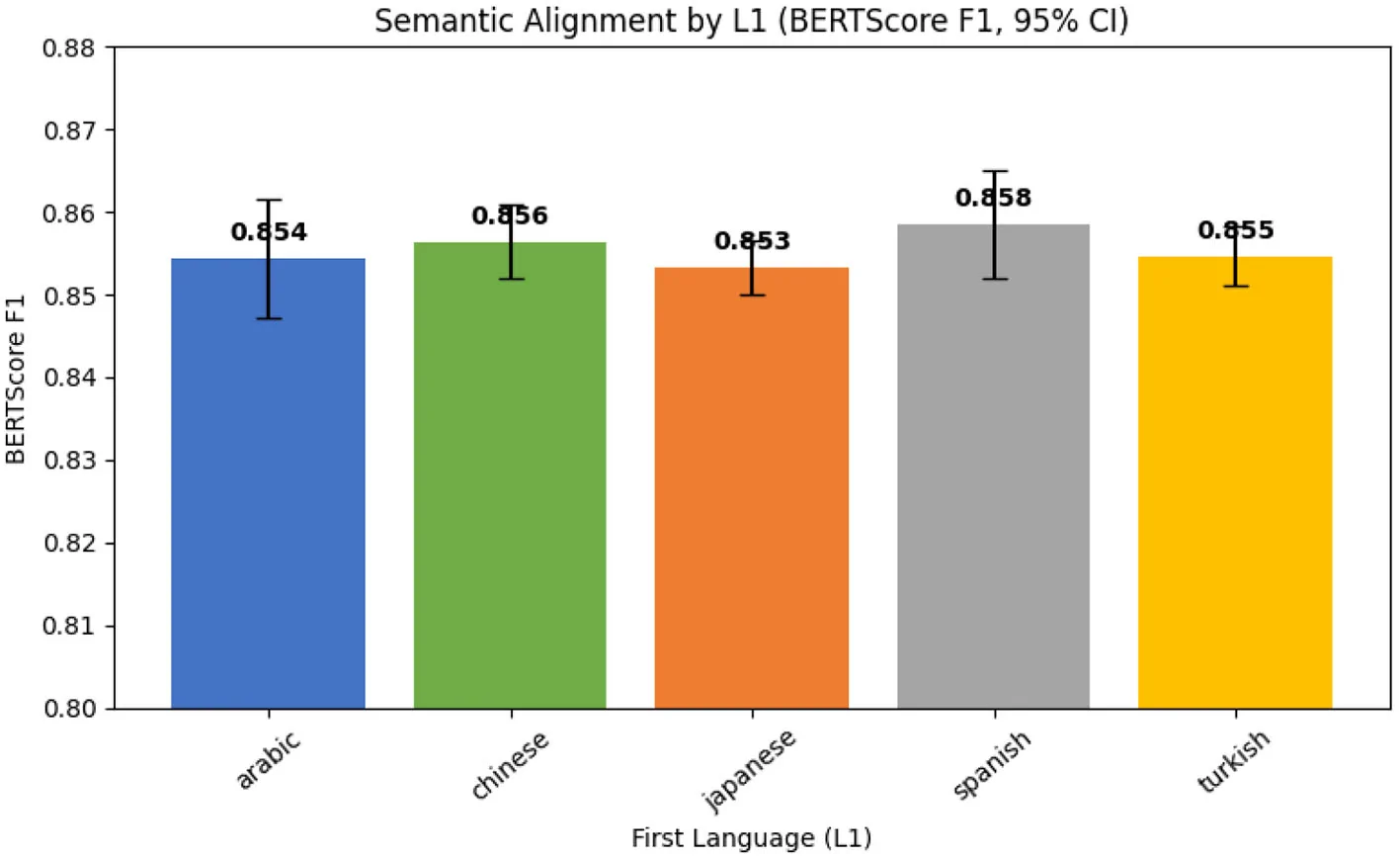
Semantic alignment by first language (BERTScore F1, 95% confidence intervals).

A similarly stable pattern was observed across proficiency levels. Mean BERTScore F1 values were 0.854 for low-, 0.856 for mid-, and 0.858 for high-proficiency learners, with no statistically significant difference across proficiency groups, H(2) = 1.388, *p* = 0.500, ε^2^ ~ 0.000. These findings indicate that the degree of semantic correspondence between GPT and simulated feedback remained broadly consistent across the L1 and proficiency groups represented in the analytical sample.

BERTScore evaluates contextual semantic correspondence using transformer-based representations rather than relying exclusively on exact lexical overlap (Zhang et al., 2019).

Accordingly, the present findings should be interpreted as evidence of semantic similarity between GPT-generated and simulated feedback, rather than as direct evidence of pedagogical accuracy, learner benefit, instructional effectiveness, or the absence of algorithmic bias.

The descriptive similarity across L1 groups was supported by the inferential analysis. A Kruskal-Wallis test found no statistically significant difference in BERTScore F1 across L1 groups, H(4) = 1.789, *p* = 0.7745, ε^2^ ~ 0.000. Similarly, BERTScore F1 did not differ significantly across proficiency levels, H(2) = 1.388, *p* = 0.4996, ε^2^ ~ 0.000. These results indicate that semantic correspondence between GPT-generated and simulated feedback was relatively stable across the L1 and proficiency groups represented in the evaluation sample.

From an equity perspective, the absence of statistically significant subgroup differences provides no evidence, within this sample, that semantic alignment systematically favored one represented L1 or proficiency group over another. However, this finding should **not** be interpreted as proof of algorithmic fairness. BERTScore measures semantic correspondence between texts rather than pedagogical effectiveness, cultural appropriateness, learner comprehension, or educational benefit. Furthermore, the relatively small and uneven subgroup sizes and the non-fully-crossed relationship between L1 and proficiency constrain the strength of fairness conclusions that can be drawn. Accordingly, the BERTScore findings are treated as one component of the broader fairness evaluation rather than as a standalone measure of bias (Blodgett et al., 2020; Baker and Hawn, 2022). Overall semantic alignment between GPT-generated and simulated feedback was high (BERTScore precision = 0.8370, recall = 0.8742, F1 = 0.8552), with no statistically significant variation in F1 across L1 or proficiency groups.

### 4.17 Comparative pedagogical characteristics

To contextualize the quantitative findings, the three feedback approaches—GPT-generated feedback, adaptive simulated feedback, and static simulated feedback—were compared across key pedagogical dimensions. Table 3 summarizes differences in feedback accessibility, elaboration, personalization, and pedagogical structure. The table distinguishes the original system-level evaluation from the verified matched-sample analysis reported in the preceding sections. Accordingly, the updated GPT length and readability statistics are based on the complete matched analytical sample (*N* = 32), whereas characteristics of the simulated systems reflect their rule-based design.

**Table 3:** Comparison of feedback approaches across key pedagogical dimensions.

Dimension	GTP feedback	Adaptive simulated feedback	Static simulated feedback
Feedback generation	Generative, context-sensitive	Rule-based, learner-sensitive templates	Rule-based/static templates
Personalization	Uses learner/context information	Predetermined adaptation rules	Limited/static
Pedagogical structure	Correction, explanation, encouragement, metalinguistic information	Structured correction and explanation	Primarily standardized correction
Mean feedback length^1^	608.12 characters	—	218.69 characters^2^
Length by proficiency^1^	Low = 657.43; Mid = 557.39; High = 602.00	—	—
Overall readability^1^	75.62	—	64.64^2^
Readability by L1^1^	Arabic = 78.04; Chinese = 78.72; Japanese = 72.22; Spanish = 73.39; Turkish = 78.92	—	—
L1 difference in length^1^	H = 6.862, *p* = 0.1434, *ε^2^* = 0.106	—	—
L1 difference in readability^1^	H = 7.547, *p* = 0.1097, *ε^2^* = 0.131	—	—
Proficiency difference in length^1^	H = 1.724, *p* = 0.4223, *ε^2^* = 0.000	—	—
Proficiency difference in readability^1^	H = 6.125, *p* = 0.0468, *ε^2^* = 0.142; no Holm-adjusted pairwise comparison significant	—	—
Evidence of proficiency adaptivity	Descriptive variation; systematic adaptation not established	Designed to vary according to predetermined proficiency rules	Limited/static
Pedagogical richness	Encouragement and metalinguistic explanation	Direct correction and structured explanation	

^1^GPT subgroup statistics were obtained from the verified matched-feedback analysis (*N* = 32).

^2^The simulated-feedback values refer to the corresponding simulated responses in the same matched analysis. Dashes indicate that a directly comparable statistic was not established in the verified analysis and is therefore not reported. Descriptive subgroup differences should not be interpreted as evidence of algorithmic bias or systematic adaptivity without corresponding inferential support.

The verified analysis indicates that GPT-generated feedback was substantially more elaborated than simulated feedback. GPT responses averaged 608.12 characters, compared with 218.69 characters for simulated responses. GPT feedback was also more readable in the matched sample, with an overall mean Flesch Reading Ease score of 75.62, compared with 64.64 for simulated feedback. Paired comparisons confirmed significant differences between GPT and simulated feedback for both length (Wilcoxon W = 0.00, *p* < 0.001) and readability (W = 22.00, *p* < 0.001).

Importantly, however, descriptive differences in GPT feedback across learner groups should not automatically be interpreted as evidence of adaptive behavior or inequity. GPT feedback length differed descriptively across proficiency levels, with means of 657.43 characters for low-proficiency, 557.39 for mid-proficiency, and 602.00 for high-proficiency learners. The overall proficiency difference was not statistically significant, H(2) = 1.724, *p* = 0.4223, ε^2^ = 0.000. Similarly, GPT feedback length did not differ significantly across L1 groups, H(4) = 6.862, *p* = 0.1434, ε^2^ = 0.106.

For readability, the L1 means ranged from 72.22 for Japanese learners to 78.92 for Turkish learners, but the overall L1 difference was not statistically significant, H(4) = 7.547, *p* = 0.1097, ε^2^ = 0.131. An omnibus proficiency difference was observed for GPT readability, H(2) = 6.125, *p* = 0.0468, ε^2^ = 0.142. However, subsequent pairwise comparisons did not remain statistically significant following Holm adjustment. These findings therefore support evidence of descriptive variation but provide limited evidence for systematic subgroup differentiation.

### 4.18 Comparative evaluation of feedback approaches

The combined analyses revealed distinct characteristics of GPT-generated and rule-based feedback. GPT responses provided substantially greater elaboration than the corresponding simulated responses and, contrary to the pattern suggested by the earlier incomplete analysis, also showed higher average readability in the verified matched sample. Semantic correspondence between GPT and simulated feedback remained stable across the L1 and proficiency groups examined.

Rule-based simulated feedback was more constrained and deterministic, but this did not result in uniform quantitative characteristics across learner groups. Significant subgroup differences were observed for simulated feedback length and readability (Table 3), reflecting variation associated with the learner-sensitive templates used to construct these responses.

The findings suggest that the distinction between generative and rule-based feedback should not be reduced to a simple trade-off between personalization and consistency. Rather, automated feedback systems should be evaluated across multiple dimensions, including explanatory detail, accessibility, semantic correspondence, and subgroup consistency. This interpretation is consistent with established research emphasizing the importance of actionable and learner-appropriate formative feedback (Hattie and Timperley, 2007; Shute, 2008).

### 4.19 System deployment and technical functionality

The Gradio-based interface was successfully deployed through Hugging Face Spaces, demonstrating the technical feasibility of delivering learner-specific spelling feedback through an interactive educational interface. The system enables users to enter a misspelled word, specify their L1 background and English proficiency level, and receive corresponding learner-oriented feedback in real time. The deployment therefore provides a proof-of-concept implementation of the proposed equity-aware tutoring framework.

Informal functional testing confirmed that the interface operated as intended in the tested environments and supported straightforward interaction with the feedback-generation components. These design characteristics are consistent with user-centered approaches to educational AI that emphasize transparent and accessible interaction between learners and intelligent systems (Holstein et al., 2019). However, because the present study did not conduct a controlled usability study involving learner participants, these observations should be interpreted as evidence of technical functionality rather than established usability, learner satisfaction, engagement, or educational effectiveness.

As illustrated in Figure 10, the system was containerized (https://huggingface.co/spaces/Oluyori/equity-ai-tutor) to support reproducibility and extensibility and incorporates spelling diagnosis, individualized feedback generation, and interaction logging. The resulting implementation provides a technical foundation for future learner-centered evaluation, longitudinal investigation of learning outcomes, and expanded fairness auditing across more diverse learner populations.

**Figure 10:**
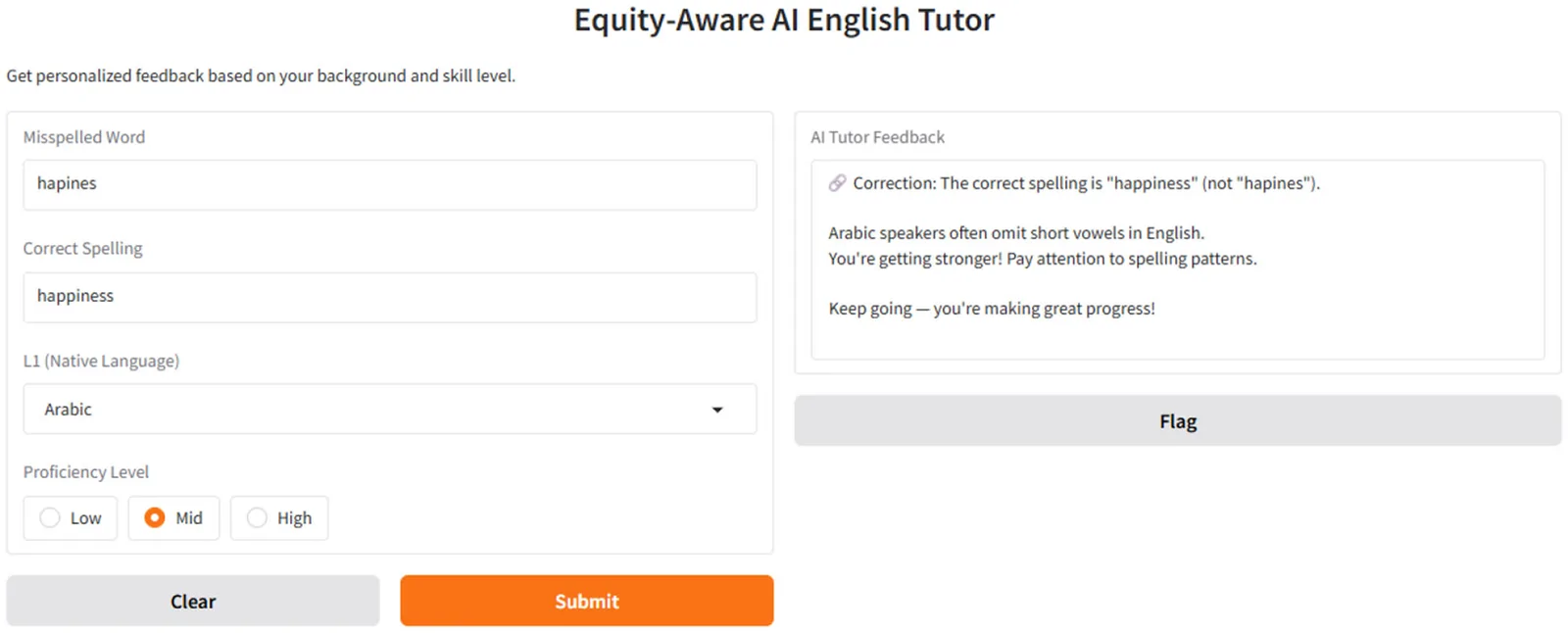
Interface of the equity-aware AI English tutor.

## 5 Discussion

Recent advances in large language models (LLMs), such as GPT-4, have created substantial opportunities for personalized feedback in computer-assisted language learning, yet questions remain regarding the pedagogical accessibility, consistency, and fairness of such systems across multilingual learner populations (Castro et al., 2024; FattahiBavandpour, 2024; Peer Mohamed, 2024). This study addressed these concerns by evaluating a GPT-supported tutoring framework in relation to linguistic accessibility, semantic alignment, feedback elaboration, learner-group variation, and fairness across first-language (L1) and proficiency groups, contributing to emerging work on equitable and explainable NLP in education (Holstein et al., 2019; Hovy and Prabhumoye, 2021). In contrast to traditional automated writing evaluation (AWE) approaches that frequently rely on rule-based or template-driven feedback mechanisms, GPT-generated feedback was substantially more elaborated while maintaining comparatively high readability. However, the corrected inferential analyses provide limited evidence that GPT systematically adapted feedback length according to learner proficiency or produced consistent L1-based differences in feedback accessibility.

Importantly, the findings also qualify the study's preliminary fairness interpretation. Although descriptive variation in feedback length and readability was observed across linguistic groups, GPT feedback length, readability, and semantic alignment did not differ significantly across L1 categories. Consequently, these differences cannot be interpreted as evidence of systemic or sociolinguistic bias on the basis of the present data alone. This distinction is consistent with broader fairness scholarship emphasizing that observed demographic disparities require consideration of data composition, learner characteristics, model inputs, and contextual factors before they can be attributed to discriminatory model behavior (Blodgett et al., 2020; Choenni, 2025; Hussain et al., 2024; Rouabhia, 2025; Sezik et al., 2025). The findings therefore position fairness auditing not as a binary determination of whether an LLM is biased, but as an ongoing assessment of whether feedback remains accessible, semantically appropriate, and pedagogically consistent across heterogeneous learner populations.

The study provides evidence that GPT can generate semantically aligned and linguistically accessible spelling feedback for multilingual learners while also demonstrating the methodological importance of distinguishing descriptive subgroup variation from statistically supported differential model behavior. Through the combined analysis of GPT-generated and rule-based simulated feedback using semantic similarity, readability, feedback-length, effect-size, and subgroup analyses, the findings illuminate both the potential and the remaining limitations of generative AI-mediated language support.

### 5.1 Pedagogical quality and semantic alignment

The semantic evaluation demonstrates substantial correspondence between GPT-generated and simulated feedback. Across the 32 matched observations, GPT-generated feedback achieved an overall BERTScore precision of 0.8370, recall of 0.8742, and F1 score of 0.8552. These findings indicate that GPT responses captured much of the semantic content represented in the corresponding simulated feedback while retaining the flexibility to generate more elaborated explanations. This result is consistent with research demonstrating that transformer-based language models can produce context-sensitive output without depending exclusively on rigid rule-based templates (Brown et al., 2020; Lin, 2024; Messer et al., 2024).

The semantic-alignment results were also notably stable across learner groups. BERTScore F1 did not differ significantly across L1 groups, H(4) = 1.789, *p* = 0.775, ε^2^ ~ 0.000, or proficiency levels, H(2) = 1.388, *p* = 0.500, ε^2^ ~ 0.000. Mean F1 values across L1 groups ranged only from approximately 0.853 to 0.858, indicating little substantive variation in semantic correspondence. From an educational perspective, this stability is encouraging because instructional feedback should preserve relevant corrective meaning regardless of learners' linguistic backgrounds. However, BERTScore measures contextual semantic correspondence rather than direct pedagogical accuracy, learner comprehension, or learning gain. The present findings therefore support semantic consistency rather than demonstrating instructional effectiveness *per se* (Zhang et al., 2019).

The results provide considerably less support for the earlier interpretation of proficiency-sensitive adaptation. GPT feedback length averaged 657.43 characters for low-proficiency learners, 557.39 for mid-proficiency learners, and 602.00 for high-proficiency learners, but these differences were not statistically significant, H(2) = 1.724, *p* = 0.422. Accordingly, response length alone cannot be interpreted as evidence that GPT systematically calibrated scaffolding according to proficiency. Although adaptive tutoring theory emphasizes differentiated instructional assistance and the learner's zone of proximal development (Chi et al., 2001; Vygotsky and Cole, 1978; Ellis, 2009; Heift and Schulze, 2007), demonstrating such adaptivity requires evidence that variation in feedback corresponds meaningfully to learners' instructional needs rather than merely to differences in generated text.

The inferential analysis likewise did not support systematic L1-based differences in GPT feedback length, readability, or semantic alignment. This finding refines the original fairness interpretation: heterogeneous outputs were observed, but the present evidence does not justify characterizing these differences as linguistic discrimination. Rather, they illustrate why descriptive differences must be tested statistically and considered alongside learner input, subgroup composition, proficiency, and error characteristics before stronger fairness conclusions are drawn.

### 5.2 Accessibility and linguistic readability

The readability findings provide important insights into the accessibility of GPT-generated feedback. Rather than being less readable than simulated feedback, GPT-generated responses achieved a significantly higher mean Flesch Reading Ease score (M = 75.62) than rule-based simulated feedback (M = 64.64). The paired difference was statistically significant, *W* = 22.00, *p* < 0.001, with a very large paired rank-biserial effect (*r* = 0.917). Thus, greater GPT elaboration did not correspond to reduced textual readability in the completed analytical sample.

This finding is pedagogically important because feedback must be sufficiently accessible for learners to process and act upon it. Readability has long been associated with the linguistic demands placed on second-language readers (Crossley et al., 2008), while excessive instructional complexity may impose unnecessary cognitive load, particularly for developing learners (Sweller, 1994). The present results suggest that GPT can generate relatively elaborate feedback without necessarily increasing surface-level reading difficulty. Nevertheless, Flesch Reading Ease measures textual properties rather than actual comprehension, and high readability should not be equated automatically with effective scaffolding or reduced cognitive load. Learner-centered comprehension studies are therefore needed before concluding that these outputs are more instructionally accessible in practice (Tran et al., 2024).

Some proficiency-related variation in GPT readability was observed. The omnibus Kruskal-Wallis test was statistically significant, H(2) = 6.125, *p* = 0.047, ε^2^ = 0.142, with mid-proficiency learners receiving the highest mean readability score (M = 78.81), compared with low- (M = 73.47) and high-proficiency learners (M = 73.39). However, none of the pairwise comparisons remained significant after Holm adjustment. The strongest contrast, between low- and mid-proficiency learners, yielded an unadjusted *p* = 0.024 but an adjusted *p* = 0.072. Consequently, the finding should be interpreted as a suggestive proficiency-related pattern rather than evidence that GPT reliably produces more accessible feedback for a particular proficiency group.

The simulated system, by contrast, demonstrated statistically significant variation in readability across both L1 and proficiency groups. This result is noteworthy because deterministic rule-based systems are often assumed to offer greater uniformity and predictability. In the present framework, however, the simulated templates explicitly incorporated learner attributes, and this predefined personalization generated systematic differences in output characteristics. Accordingly, transparency alone does not guarantee uniform accessibility; both generative and rule-based educational systems require careful calibration and evaluation (Heil et al., 2016; Warschauer and Matuchniak, 2010; Warschauer et al., 2023).

### 5.3 Equity and algorithmic fairness

The fairness analysis provides a more cautious and empirically grounded account of learner-group variation. GPT feedback length did not differ significantly across L1 groups, H(4) = 6.862, *p* = 0.143, ε^2^ = 0.106, and GPT readability similarly showed no statistically significant L1 difference, H(4) = 7.547, *p* = 0.110, ε^2^ = 0.131. Semantic alignment was particularly stable, with no significant L1-related difference in BERTScore F1, H(4) = 1.789, *p* = 0.775. These findings do not support the preliminary conclusion that Arabic or Chinese learners were systematically privileged relative to Japanese, Spanish, or Turkish learners.

This distinction is important in light of established concerns regarding representational and performance disparities in NLP systems (Achiam et al., 2023; Blodgett et al., 2020). Prior work has shown that imbalanced training corpora and uneven linguistic representation can contribute to differential system behavior (Zhao et al., 2017); however, the existence of such risks does not mean that every observed group difference constitutes algorithmic bias. Fairness assessment therefore requires attention to the mechanism generating the disparity, its magnitude, its statistical reliability, and its educational consequence (Holstein et al., 2019; Hovy and Spruit, 2016; Baker and Hawn, 2022).

The controlled sensitivity analyses further support a cautious interpretation. After accounting for prompt length, misspelling length, and spelling-length difference, the analyses provided no consistent evidence that L1 independently predicted GPT readability or semantic alignment. Although one Japanese coefficient emerged in the feedback-length model, the overall L1 model did not reach statistical significance and the result should therefore not be interpreted independently as evidence of L1-specific disadvantage.

The sample structure imposes an additional constraint on fairness interpretation. L1 and proficiency were not fully crossed: Arabic and Japanese observations occurred within the low-proficiency category, Chinese and Turkish observations within the mid-proficiency category, and Spanish observations within the high-proficiency category. Consequently, L1- and proficiency-associated variation cannot be completely disentangled. The present fairness analysis therefore identifies fairness-relevant patterns and tests their statistical robustness but does not establish causal discrimination.

These findings refine rather than diminish the importance of fairness-aware educational AI. They demonstrate that fairness evaluation should extend beyond simple equality of response length or readability and should instead examine whether systems provide comparably meaningful, accessible, and pedagogically appropriate support across learner populations. Such an approach is consistent with broader calls for culturally responsive and linguistically inclusive AI design (Blodgett et al., 2020; Holstein et al., 2019; Hovy and Spruit, 2016).

### 5.4 Toward hybrid feedback systems

The findings continue to support the value of hybrid feedback architectures, although the rationale differs somewhat from the preliminary interpretation. GPT-generated feedback demonstrated substantial semantic alignment, substantially greater elaboration, and higher average readability than the corresponding simulated feedback. Rule-based feedback, meanwhile, offered transparent and deterministic generation procedures but exhibited significant subgroup variation in both length and readability. Thus, the results do not support a simple distinction in which GPT provides richness at the expense of accessibility while templates provide uniformity and clarity. Instead, each approach offers different strengths and constraints.

A hybrid system remains attractive because generative models can provide contextualized explanation, encouragement, and flexible linguistic realization, whereas rule-based mechanisms can impose explicit pedagogical constraints and safeguard required instructional content (Hattie and Timperley, 2007; VanLehn, 2011). Such safeguards may be used to establish minimum feedback requirements, constrain unnecessary complexity, ensure that key corrections are consistently communicated, and monitor accessibility without eliminating the generative flexibility of LLMs. This interpretation is consistent with recommendations for educational AI systems that combine computational adaptivity with transparent pedagogical structures (Holmes et al., 2019).

The simulated pipeline developed in this study, incorporating L1-aware heuristics and proficiency-sensitive templates, provides a practical foundation for this form of hybridization. At the same time, its own significant subgroup variation demonstrates that rule-based personalization must also be audited rather than assumed to be inherently equitable. Fairness therefore becomes a property of the complete instructional system, including data, rules, prompts, learner attributes, and output constraints, rather than a property unique to the generative model.

The Gradio-based implementation further provides a practical environment for iterative inspection of generated feedback and future learner-centered testing. Such interactive deployment can support systematic auditing, educator evaluation, and subsequent refinement of instructional constraints, consistent with broader principles of user-centered educational technology design (Luckin and Holmes, 2016). Future implementations could incorporate dynamic readability targets, explicit instructional templates, human-in-the-loop review, multilingual adaptation, and fairness-monitoring mechanisms to improve consistency across learner populations.

The results support hybridization not because GPT necessarily produces inaccessible or systematically biased feedback, but because combining generative flexibility with transparent instructional safeguards may provide a more controllable and pedagogically accountable approach to AI-mediated feedback.

### 5.5 Theoretical and practical implications

Theoretically, this study contributes to educational technology, applied linguistics, and fairness-aware NLP by demonstrating that learner-sensitive AI feedback must be evaluated not only in terms of generated content but also in terms of accessibility, semantic consistency, subgroup variation, and evidentiary strength. The inferential analysis did not support the interpretation that variation in feedback length represented systematic proficiency-sensitive scaffolding. This finding is theoretically important because adaptive behavior should not be inferred merely from heterogeneous model output. Instead, claims of differentiated instruction should demonstrate that system responses vary systematically and appropriately according to learner needs, consistent with scaffolding and Zone of Proximal Development perspectives (Vygotsky and Cole, 1978; Nassaji and Kartchava, 2017; Tomlinson, 2017).

The study also contributes a more conservative fairness framework for multilingual educational AI. The absence of statistically significant L1 differences in GPT length, readability, and semantic alignment suggests that descriptive variation should not automatically be characterized as linguistic bias. At the same time, the study does not establish that GPT is universally fair; rather, it demonstrates the importance of combining subgroup diagnostics with statistical testing, effect-size estimation, input-level controls, and careful consideration of sample structure. This approach is consistent with wider concerns about responsible interpretation of algorithmic disparities in language technologies (Blodgett et al., 2020; Hovy and Spruit, 2016).

Practically, the study provides a deployable framework for learner-oriented spelling feedback through Hugging Face Spaces. GPT-generated feedback was substantially longer and more readable than simulated feedback in the verified matched sample, suggesting that generative explanations can offer considerable detail without necessarily sacrificing surface-level accessibility. However, because readability does not directly measure learner comprehension or instructional benefit, educators and developers should incorporate learner testing rather than relying exclusively on automated metrics.

The findings also emphasize the importance of auditing rule-based systems. Significant L1- and proficiency-associated differences in simulated feedback demonstrate that deterministic personalization can itself create systematic output variation. Developers should therefore evaluate both generative and template-based components for accessibility and consistency rather than assuming that one architecture is intrinsically more equitable. Hybrid systems incorporating explicit instructional constraints, readability monitoring, and educator oversight may provide a productive direction for responsible deployment.

## 6 Limitations and future work

The findings should be interpreted in light of several methodological and analytical constraints. The study focused on spelling feedback derived from the TOEFL-Spell corpus and therefore captures only a limited subset of the challenges encountered in second-language writing. Grammatical, syntactic, discourse-level, and rhetorical dimensions were outside the scope of the present analysis, which limits the extent to which the findings can be generalized to broader forms of automated writing support or to learner populations beyond those represented in the corpus.

The inferential evaluation was based on 32 complete GPT-simulated feedback pairs. Although completion of the feedback-generation pipeline removed missing-output artifacts and enabled a more rigorous statistical comparison, the overall sample remained modest and several L1 subgroups were small. This necessarily reduces statistical power and increases uncertainty around subgroup estimates, particularly for pairwise comparisons and exploratory regression analyses. The structure of the sample also imposed an additional constraint because L1 and proficiency were not fully crossed. Arabic and Japanese observations were represented within the low-proficiency category, Chinese and Turkish observations within the mid-proficiency category, and Spanish observations within the high-proficiency category. As a result, L1- and proficiency-associated patterns cannot be interpreted as fully independent effects. Although the controlled analyses incorporated prompt length, misspelling length, and spelling-length difference, these adjustments cannot replace a balanced factorial design in which each linguistic group is represented across multiple proficiency levels.

A further limitation concerns the evaluation metrics themselves. Feedback length, Flesch Reading Ease, and BERTScore provide useful information about elaboration, surface-level linguistic accessibility, and semantic correspondence, but they do not directly measure whether learners understand the feedback, find it helpful, revise more successfully, experience reduced cognitive load, or achieve measurable learning gains. BERTScore, in particular, quantifies semantic alignment between GPT-generated and simulated feedback rather than independently validated pedagogical accuracy. The absence of direct learner and educator evaluation therefore limits claims about instructional effectiveness, usability, and cultural appropriateness.

These limitations point to several priorities for future research. Larger and more balanced multilingual samples would allow L1 and proficiency effects to be examined independently and would strengthen both subgroup comparisons and fairness analyses. Extending the framework beyond spelling to grammatical, syntactic, discourse-level, and rhetorical feedback would also improve its ecological validity and establish whether the observed patterns generalize across broader writing tasks. Learner-centered and educator-centered evaluation should be incorporated to assess comprehension, perceived helpfulness, revision quality, engagement, and longitudinal writing development, thereby complementing automated metrics with direct evidence of pedagogical value.

Future work should also broaden the operationalization of fairness beyond response length and readability. Measures of correction accuracy, feedback helpfulness, error severity, cultural appropriateness, consistency, and learning outcomes could provide a more comprehensive account of whether AI-mediated feedback supports multilingual learners equitably. Multilingual fine-tuning, dynamic readability calibration, educator co-design, explainable AI mechanisms, and human-in-the-loop review may further strengthen the pedagogical reliability and transparency of the system. Together, these extensions would help distinguish appropriate personalization from random model variation or systematic educational disadvantage and would provide a stronger empirical basis for the responsible deployment of generative AI in multilingual language learning.

## 7 Conclusion

This study developed and evaluated a fairness-aware AI tutoring framework for providing spelling feedback to multilingual English learners through GPT-generated and rule-based simulated responses. The completed analysis demonstrates that GPT-generated feedback achieved substantial semantic alignment with simulated feedback (BERTScore precision = 0.8370, recall = 0.8742, F1 = 0.8552) while producing substantially longer and, on average, more readable responses. GPT feedback length, readability, and semantic alignment did not differ significantly across L1 groups, while an omnibus proficiency-related difference in readability did not yield statistically significant pairwise contrasts after correction for multiple comparisons.

These findings refine the study's preliminary fairness interpretation. Rather than demonstrating systematic L1-based linguistic bias, the results indicate descriptive variation in learner-facing feedback that was not consistently supported as statistically significant differential model behavior. At the same time, the findings should not be interpreted as proof of algorithmic fairness because subgroup sizes were small, L1 and proficiency were partially confounded, and the evaluation focused on a limited set of computational indicators. The study therefore demonstrates the importance of distinguishing observable subgroup variation from evidence of systematic inequity when evaluating generative educational systems (Blodgett et al., 2020; Hovy and Spruit, 2016).

The comparison with rule-based feedback further showed that deterministic systems are not necessarily uniform across learner groups, particularly when personalization rules explicitly incorporate learner characteristics. This finding strengthens the case for auditing the full educational AI pipeline rather than focusing exclusively on the generative model. Hybrid architectures that combine LLM-generated explanations with transparent instructional safeguards, accessibility monitoring, and learner-centered evaluation may therefore offer a productive pathway toward responsible AI-mediated language instruction.

This work contributes to equity-centered educational AI by integrating multilingual learner information, systematic subgroup evaluation, semantic-alignment analysis, accessibility assessment, and interactive deployment within a unified tutoring framework. It also advances a more methodologically cautious approach to fairness evaluation in which claims of algorithmic bias are supported by inferential evidence rather than descriptive differences alone, responding to broader calls for interdisciplinary and responsible approaches to educational AI (Chinta et al., 2024; Ercikan and Por, 2020).

## Data Availability

The original contributions presented in the study are included in the article/Supplementary material, further inquiries can be directed to the corresponding author.
