# Supplementary material for: Fairness-aware artificial intelligence tutoring for multilingual learners: evaluating adaptive feedback across L1 and proficiency groups
Source: Front Artif Intell. 2026 Sep 22;9:1934329. doi: 10.3389/frai.2026.1934329 (PMC13638495; doi:10.3389/frai.2026.1934329)
Supplement: Supplementary file 1 [file Supplementary_file_1.docx]

**Appendix**

**Prompt**

Tutoring Prompt:

You are an AI tutor helping a multilingual English learner improve their spelling.

Essay excerpt:

"""

There is a saying in my language that goes like: 'If only the young could know and the old could do'. ...

While you're young your mind is fresh, open for ideas and future is full of possibilties. ...

Excitement is more preferable than the regret which inevitably comes with the old age and even feeling of fullfillment can't beat the <<hapiness>> you feel when you're upon a new discovery. ...

"""

The student wrote: "hapiness"

The correct spelling is: "happiness"

Please explain the mistake kindly. Include:

- The correct spelling

- A memory tip or rule

- Words of encouragement

Response:

Highlighted Essay:

There is a saying in my language that goes like: 'If only the young could know and the old could do'. ...

While you're young your mind is fresh, open for ideas and future is full of possibilties. ...

Excitement is more preferable than the regret which inevitably comes with the old age and even feeling of fullfillment can't beat the <<hapiness>> you feel when you're upon a new discovery. ...

LLM Prompt:

You are an AI writing tutor helping a multilingual student improve their English.

Essay excerpt:

"""There is a saying in my language that goes like: 'If only the young could know and the old could do'. This explains an important lesson, but one has to attain a certain degree of wisdom to understand it. In my opinion being young is more enjoyable, being older may make somebody more experienced but it would not make his life less boring.

While you're young your mind is fresh, open for ideas and future is full of possibilties. Excitement is more preferable than the regret which inevitably comes with the old age and even feeling of fullfillment can't beat the <<hapiness>> you feel when you're upon a new discovery. Just remember your greatest triumph. Would you feel the same after ten years passed?

Nobody can deny that having an energetic, heathy body is better than being frail, dependant on medicine or on someone. You can't try parachute jumping if you're past 60. Doctors wouldn't allow it. Even the eating wouldn't be the same with lots of constraints.

It's always easier to meet and get on well with new people as a young person. People accept and trust each other easily. It's simple to find common ground when you're young. Older people tend to be more rigid in their views. Traits and ideas begin to turn to stone. After a certain age it becomes impossible to change them.

Let's face it; people don't get wiser when they get old. They only get old, both in mind and the body. It's becomes harder and harder to deal with them so unfortunatly some families even consider to send them away.

"""

The student wrote: "hapiness"

The correct spelling is: "happiness"

Please explain the mistake gently and clearly. Include:

- The correct spelling

- A helpful tip (like a memory rule)

- Words of encouragement
